# Caribbean cyclone activity: an annually-resolved Common Era record

**DOI:** 10.1038/s41598-020-68633-8

**Published:** 2020-07-16

**Authors:** Dominik Schmitt, Eberhard Gischler, Flavio S. Anselmetti, Hendrik Vogel

**Affiliations:** 1 Institut für Geowissenschaften, Johann Wolfgang Goethe-Universität, Altenhöferallee 1, 60438 Frankfurt, Germany; 2 Institut für Geologie & Oeschger Centre for Climate Change Research, Universität Bern, Baltzerstraße 1+3, 3012 Bern, Switzerland

**Keywords:** Palaeoceanography, Palaeoclimate, Natural hazards, Physical oceanography

## Abstract

Tropical cyclones (TC) represent a substantial threat to life and property for Caribbean and adjacent populations. The prospective increase of TC magnitudes, expressed in the 15th chapter of the IPCC AR5 report, entails a rising probability of ecological and social disasters, which were tragically exemplified by several severe Caribbean TC strikes during the past 20 years. Modern IPCC-grade climate models, however, still lack the required spatial and temporal resolution to accurately consider the underlying boundary conditions that modulate long-time TC patterns beyond the Instrumental Era. It is thus necessary to provide a synoptic mechanistic understanding regarding the origin of such long-time patterns, in order to predict reliable changes of TC magnitude and frequency under future climate scenarios. Caribbean TC records are still rare and often lack the necessary continuity and resolution to overcome these limitations. Here, we report on an annually-resolved sedimentary archive from the bottom of the Great Blue Hole (Lighthouse Reef, Belize). The TC record encompasses 1885 years and extends all existing site-specific TC archives both in terms of resolution and duration. We identified a likely connection between long-term TC patterns and climate phenomena responses to Common Era climate variations and offer a conceptual and comparative view considering several involved tropospheric and oceanographic control mechanisms such as the El-Niño-Southern-Oscillation, the North Atlantic Oscillation and the Atlantic Multidecadal Oscillation. These basin-scaled climate modes exercise internal control on TC activity by modulating the thermodynamic environment (sea-surface temperature and vertical wind shear stress dynamics) for enhanced/suppressed TC formation both on millennial (primary) and multi-decadal (secondary) time scales. We interpret the beginning of the Medieval Warm Period (MWP) as an important time interval of the Common Era record and suspect that the southward migration of the intertropical convergence zone (ITCZ) caused, in combination with extensive hydro-climate changes, a shift in the tropical Atlantic TC regime. The TC activity in the south-western Caribbean changed in general from a stable and less active stage (100–900 CE) to a more active and variable state (1,100 CE-modern).

## Introduction

Tropical cyclones (TC) generally develop over tropical Atlantic areas within the trade-wind zone. The Atlantic cyclone main development region (MDR) is characterised by a latitudinal extension from 9° N to 20° N and runs longitudinally along the northern edge of the intertropical convergence zone (ITCZ)^1^. Sea-surface temperatures (SST) exceeding the threshold of 26.5 °C, the influence of low vertical wind shear stress, and the occurrence of steady vertical (tropospheric) temperature gradients are the major natural boundary conditions for TC formation^2^. These boundary conditions facilitate a deep atmospheric convection within the tropical western Atlantic and exercise thus a thermodynamic control on TC activity. Instrumental data (NOAA HURDAT2), case studies^3^ and global warming prediction models^4^ generally agree that the magnitude of TCs will increase under future greenhouse-climate conditions, whereas the average frequency of the different TC categories will remain the same or indeed slightly decrease^5^. In order to reconcile and specify these trends, other prediction models^6,7^ demonstrate a shift towards more frequently occurring very intense TCs (doubling of cat.4/5 hurricanes) and a related decline in the frequency of weaker storms. MDR SST variations and coevally occurring changes of vertical wind shear stress dynamics have been successfully identified as the primary TC activity control factors for the short instrumental record^8^. In addition to the atmospheric modulation in response to the El-Niño-Southern-Oscillation (ENSO), SST variations are therefore a further key factor to understand future, present and past patterns of TC activity^9^. There is a contested theory that the Atlantic Multidecadal Oscillation (AMO) is a major control factor for the North Atlantic climate variability^10^ and multi-decadal variability in Atlantic SST^11^. The AMO mode is attributable to 40–70 years long periodic cycles originating from velocity fluctuations of the thermohaline circulation (THC)^12^. The interplay of accelerated/decelerated THC and concurrent positive/negative AMO phases exerts control on ocean heat transfer rates between the tropical (0°) and Northern Atlantic (60°), which in turn induces multi-decadal fluctuating SST anomalies^12^. This connection of SST variability and AMO control could be, however, also a potential long-term driver of TC activity patterns^13^. The North Atlantic Oscillation (NAO) is another climate driver, which was previously considered as an important atmospheric phenomenon influencing the tracking of TCs by North Atlantic atmospheric pressure gradient modulations^14^. These air pressure gradient variations between the Icelandic Low and the Azores High are also associated with the creation of a thermodynamic environment for enhanced/suppressed TC formation as response to higher/lower heat transfer rates and lower/higher tropospheric wind shear stress^15^. This linkage has been previously used as a major climatic variable to explain on centennial scales a higher south-western Caribbean TC activity during the Medieval Warm Period (NAO+) and a lower TC activity during the Little Ice Age (NAO−)^16^. The NAO is, however, primarily active during the boreal winter, a time when Atlantic TC formation does not occur. This issue raises questions about the suitability of the NAO mode as a major TC activity control factor. The NAO may be therefore better suited to explain long-term changes in the Atlantic hydro-climate and should be regarded as one part of the entire system, but not as the primary control factor shaping TC activity on longer time scales as suggested in a former site-specific study^16^. In contrast, it is crucial to understand the long-term role of the AMO mode within this positive feedback system. A long-term consideration of the AMO phenomenon enables a coupled consideration of centennial-scaled SST variations and long-term atmospheric ENSO modulations. Both climate modes are certainly more appropriate to explain long-term patterns of Common Era TC activity than the previously assumed NAO connection. The role of the two ENSO phases (El-Niño/La-Niña) for modulating the Atlantic TC activity is relatively well understood considering multi-annual observational time-series^17^. During El-Niño years, fewer Atlantic TCs develop due to stronger influence of vertical wind shear stress, stronger trade winds and an overall greater atmospheric stability. Weaker vertical wind shear stress, weaker trade winds and a lower atmospheric stability favour in turn a higher TC activity within La-Niña years. The positive feedback between AMO, ENSO and TC activity modulation, is in particular on longer-time scales not fully understood and as described above mainly limited to the Instrumental Era record, a tree ring based AMO reconstruction^18^ and a spatial grid study in the western Caribbean region that focusses on a coupled ENSO and AMO influence for shaping boundary conditions of TC formation^19^. The AMO related uncertainty in former TC activity reconstructions might partly originate from the fact that it is not entirely clear how far back in time the AMO phenomenon occurs and how precisely this climate driver acts together with the ENSO dynamics to shape TC activity patterns on longer than multi-decadal time scales. It is essential to produce sensitive and temporarily continuous TC activity records with annual resolution and reliable AMO signal detection, to overcome this limitation and to provide a thorough understanding of all involved climate modes.

Continuous TC activity records with annual resolution are still rare, often incomplete (duration and spatial resolution) and indicate, in addition, conflicting results in terms of long-term Caribbean activity patterns. Some discontinuous TC records have been obtained from over-washed sand layers, storm rubble and boulder ridges above highest astronomical tides, or from the onshore occurrence of marine organisms within coastal lagoons of Belize^20^ and Puerto Rico^21^ as well as in the coastal Belize marshes^22,23^. These studies reach only a decadal resolution and assume constant sedimentation rates which is, however, not representative, because of erosional unconformities in the stratigraphic record. Annual resolution records are in general the exception in coastal environments (e.g. Salt Pond, Massachusetts^24^).

**Figure 1:**
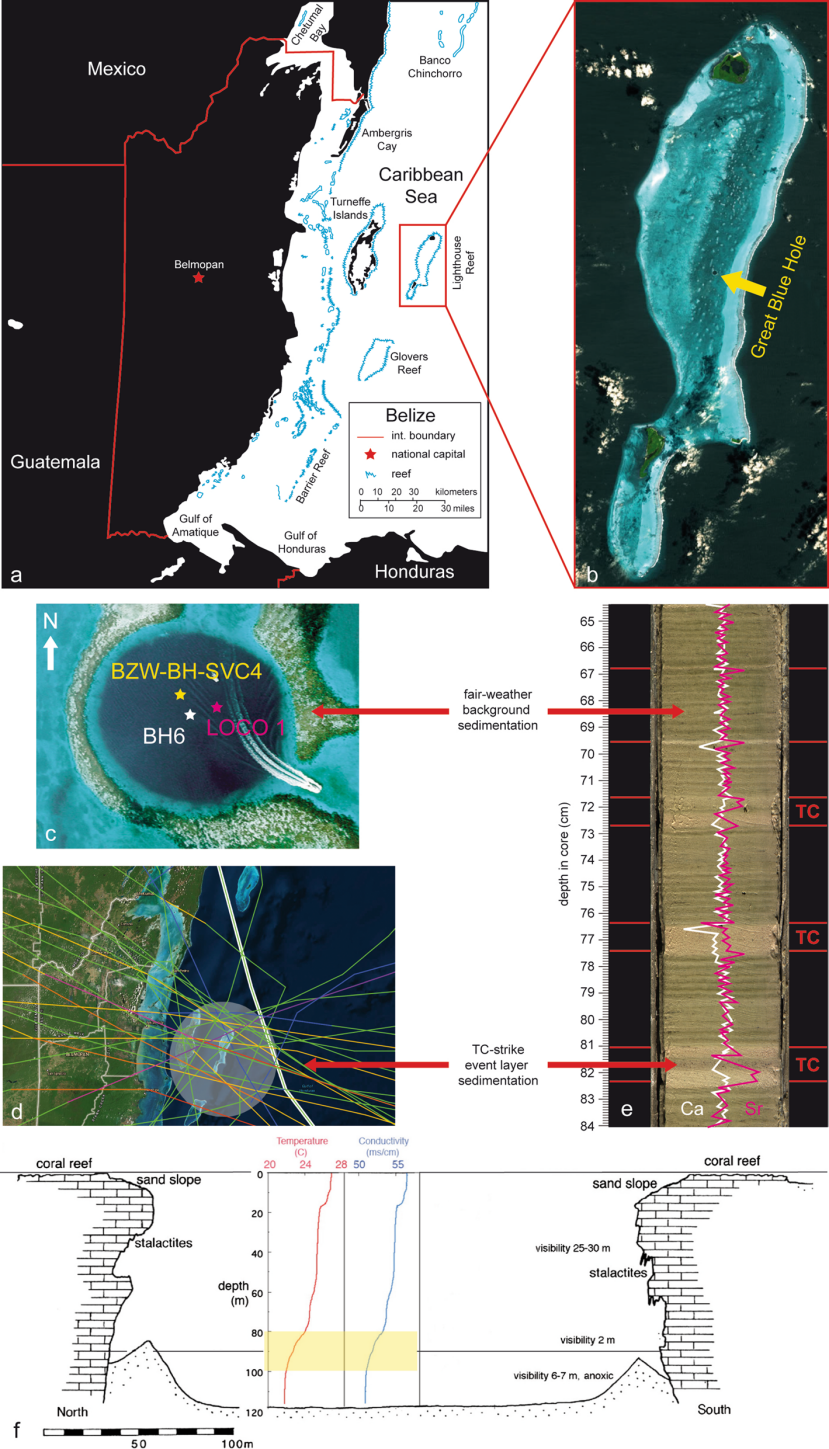
(**a**) Geographical overview illustrating the position of Belize and offshore atolls in south-western Caribbean area. The map was produced digitizing open access satellite images using the vector graphic software Adobe Illustrator CS4 V.14.0. We acknowledge the use of imagery from the NASA Worldview application (https://worldview.earthdata.nasa.gov), part of the NASA Earth Observing System Data and Information System (EOSDIS). (**b**) Open access ASTER satellite image of Lighthouse Reef (https://asterweb.jpl.nasa.gov) supplied by Japan Space Systems, and U.S/Japan ASTER Science TEAM (NASA/METI/AIST/). The Great Blue Hole is situated in the eastern lagoon of Lighthouse Reef. (**c**) Aerial view taken in a previous study^28^ indicating the position of the core BH6 (17° 18.957′ N, 87° 32.098′ W) in the Great Blue Hole centre as well as cores obtained during previous studies (BZE-BH-SVC4^16^ and LOCO1^28^). (**d**) Historical TC record (1,850–2,017 CE) passing the study area within a hypothetical 100 km diameter circle (grey shaded). The cyclone track map was downloaded from https://coast.noaa.gov/hurricanes/ and compared with NOAA HURDAT dataset^29^. (**e**) Core photo (interval BH1: 65.0–84.0 cm) of the undisturbed sediment succession at the floor of the Great Blue Hole comprising fine-grained, yellow–brown/green laminated couplets and five intercalated coarser sandy event beds (red). Additionally, Sr (purple) and Ca (white) overlays of XRF-datasets (elemental intensity: counts per second = cps) are included, illustrating the approach of Sr/Ca ratios (relative variation to background) for TC detection. (**f**) The Great Blue Hole cross-section with temperature and conductivity profiles^28^. Temperature and conductivity decrease significantly between 80 and 100 m depth; bottom water below ca. 90 m is anoxic^30^.

Blue Holes are indeed ideal sites for both annually resolved and continuous long-term TC reconstructions, because they are not affected by erosional unconformities or other geomorphological processes related to changes of accommodation space, sediment supply and subsidence, as it is the case in coastal settings. There are some examples of TC records obtained from Blue Hole depositional settings with multi-annual (e.g. the Florida Mullet Pond record^25^) and multi-decadal resolution (e.g. the Bahamas Blackwood^1^ and Thatchpoint sinkhole records^26^), respectively. The most recent study on Southern Andros Blue Holes (Bahamas)^27^ actually reaches annual resolution. Our study investigates the Great Blue Hole of Lighthouse Reef (Belize), which is an inundated, circular and cylindrical-shaped Pleistocene karst cavity acting as sediment trap in the wake of multiple historical TC tracks (Fig. 1a,b,d). The same sinkhole has already been sampled before with cores covering the past ca. 1,380 years and 2–4 years resolution^16,28^. The sedimentary archive of the Great Blue Hole offers, however, the great potential to be analysed further back in time and at annual resolution.

We cored the 8.55 m long record (BH6) at 17° 18.957′ N, 87° 32.098′ W in the approximate centre of the sinkhole, in August 2017 (Fig. 1c). The core stratigraphy is bipartite (Fig. 1e) and characterized by yellow/brown-greenish, varved fair-weather background sedimentation and yellow/brown storm-induced event layers with varying amounts of skeletal debris (*Halimeda*, coralline red algae, molluscs, foraminifers, corals, echinoderms) and organic particles^31,32^. The annual couplets most likely reflect seasonal changes in primary productivity within the surface waters of the Lighthouse Reef lagoon, resulting in differences in organic matter content between summer (yellow/brown) and winter season (green)^28^. The varved background sedimentation layers are perfectly preserved in the sedimentary subsurface of the Great Blue Hole and totally undisturbed from bioturbation, because of persistent anoxic bottom-water conditions (Fig. 1f). TCs passing the Great Blue Hole sediment trap either cause a sediment slope collapse at the top of the sinkhole, or physically initiate a suspension and settling of coarse-grained carbonate particles (> 63 μm) from the surrounding lagoon floor and marginal reef through storm wave and surge impact^33^. The coarser and sometimes graded event layers reveal both sharp erosive (downslope density flows) and gradual contacts (settling out of suspension) to the varved section^33^. The gradation trends are generally comparable to the textural behaviour of a classic storm surge deposit. Many storm layers consist of three storm-driven subsections of changing energy levels indicated by mean grain size variation: onset (slightly increased grain size against background deposits), maximum (abrupt increase in grain size) and abatement (decline in grain size) of TC landfall.

Event layers have been generally identified in previous studies on the same sinkhole^16,28^ based on colour difference, layer thickness or grain-size estimation. The grain-size estimation, in particular, conveys the impression that all sediment layers in the Great Blue Hole exceeding 30 μm are generally attributable to event beds. However, there remain in detail some crucial limitations concerning this general grain-size threshold. It is for example particularly important to consider that “lobe-like” event layer geometry causes variations in layer thickness and grain sizes at different coring sites. Core-specific grain-size measurements of the background sedimentation are essential requirements for creating a grain-size related TC identification approach. The background sedimentation in core BH6 differs from previous thresholds and was determined to have an average grain size of 20 +/− 4 μm. All measured grain-size values exceeding this threshold have to be compared in the same core to grain-size values of historical record TCs and thus calibrated and verified. This calibration step allows, however, only a limited use of grain-size values in the BH6 core, because of grain size variations along the core originating from different hydrodynamic characteristics of various reefal carbonate particles. These limitations have large effects on the interpretation of sediment texture and of course hamper a pure TC identification on basis of average core background sedimentation grain-size. The integration of high-resolution XRF-scanning datasets (> 20,000 samples) is a new valuable TC identification tool usable in sinkholes characterized by calcitic background sedimentation. XRF data are sensitive to record changes in background (containing calcite) versus over-wash (rich in aragonitic reef fragments) sediments. In particular, peaks in the Sr/Ca-ratio are very suitable to identify event layers in carbonate environments owing to the enrichment of Sr in aragonite relative to calcite (Fig. 1e). We applied a multi-proxy-approach to ensure a more reliable TC identification (Suppl. 1) addressing the above-mentioned limitations. Our quantitative approach requires at best match to fulfil five different event layer identification criteria: (1) grain-size > 20–24 μm; (2) < 85% of fine material < 63 μm; (3) layer-thickness > 2.5 mm; (4) yellow/brown colour and (5) Sr/Ca-ratio > 0.025.

**Figure 2:**
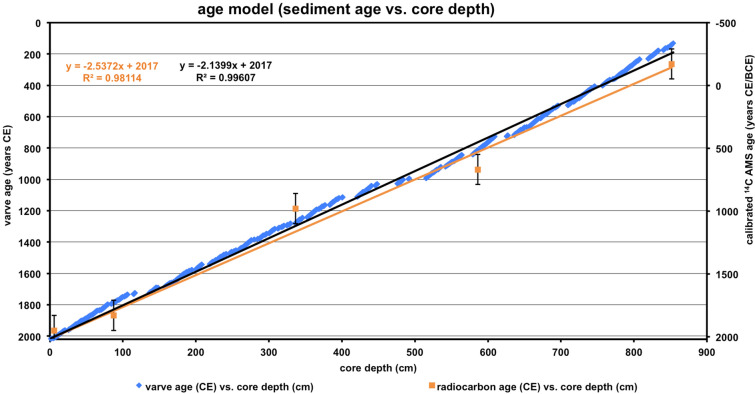
Combined plot of sediment age (years CE) against core depth (cm) as determined by both calibrated ^14^C AMS radiocarbon geochronology of organic matter residue and by varve counting off buff-green background sediment couplets. Event layers have been omitted in our age model (gaps).

The chronology of individual TC strike event layers in core BH6 is anchored on two well-established and independent age/depth frameworks (Fig. 2). For this we use a combination of radiocarbon and varve age-models. Both models explicitly show a very strong linear correlation (r^2^ = 0.9) without any significant age offset between the counts of annual green-buff couplets and five ^14^C ages obtained by AMS dating of entirely marine organic residue (δ^13^C_org_average_ =  − 16.0 ‰). As such, our age model allows reconstructions of continuous TC activity with annual resolution back to the year 130 CE.

The presented multi-proxy-approach combines classical storm-layer detection methods with improved quantitative textural analyses and innovative XRF scanning applications. This proceeding enables, in combination with an annual resolution age-model (sedimentation rate of 2.5 mm/year; sampling interval of 2.5 mm), a so far unmatched Common Era evaluation of south-western Caribbean TC frequency. TC counts and textural parameters have been further investigated using multivariate statistics, in order to decipher possible temporal patterns and statistically significant correlations with climate phenomena such as the AMO and ENSO^34,35^.

### TC record: quality and proxy results

We compared the occurrence of event layers in the core BH6 with the historical record of TCs passing Lighthouse Reef within a 100 km radius to assess the detection sensitivity of the record (Suppl. 2). The TC archive of Lighthouse Reef is generally sensitive to all storm categories. The site has, however, like many other TC archives, a minimum bias of 10% for undetected storm events. In total, 30 TCs (intensity: tropical depression to major hurricane) passed Lighthouse Reef since 1,850 CE. Of these, 21 TCs were matched with detected event layers near the anticipated sediment age of the historical strike year of the storm system, considering age offsets from varve counting of +/− 1 year. Nine storm systems from the historical record could not be observed in the BH6 core near the expected depth and varve age. Among these are four tropical storms (Kyle 1996 CE, Gert 1992 CE, Laura 1971 CE, Gilda 1954 CE), three hurricanes of category 1–3 (Edith 1972 CE, Abby 1960 CE, Greta 1978 CE) and two major hurricanes of category 4–5 (Keith 2000 CE, Mitch 1998 CE) using the 5-point-Saffir-Simpson scale. Hurricane Mitch (1998 CE) remains probably undetected in the record, because of its untypical southward migration and large catchment area distance (not enough wave energy to produce an event layer). The lack of a storm layer from Hurricane Keith (2000 CE), which directly moved over Lighthouse Reef, remains largely enigmatic. The missing event layer could probably be the result of a spatially limited density-surge deposition. Apparently, event-layer geometry in the Great Blue Hole has in many cases the shape of lobes that do not cover the entire bottom of the sinkhole. The remaining four tropical storms and three hurricanes of category 1–3 have been identified in another Great Blue Hole core with a detection uncertainty of 20%^16^. This comparison illustrates the challenges in high-resolution paleotempestology and the need of several comparable cores from the same study site. We must admit a 30% average bias for underrated TC activity in the BH6 core by missing nine out of 30 historical record storm systems. The general bias of the Great Blue Hole for undetected storm events decreases down to 7% combining two site-specific cores (BH6 and BZW-BH-SVC4^16^) and their individual historical record calibrations. Eight additional event layers did not match any known historical TC-events since 1,850 CE. Some of the additional “non-TC layers” of the Instrumental Era may be caused by the fact that the study site is situated next to an active strike-slip zone along the Caribbean and North American plate boundary. Seismic events may also trigger instabilities on the sediment slope located at the top of the Great Blue Hole and cause sediment redeposition to the bottom of the sinkhole, thereby leaving a coarser-grained event bed. Two “non-TC layers” (2,004 CE and 2,017 CE) are probably correlated with strong seismic events adjacent to the study area. These include two earthquakes near the Swan Islands (2,004 CE) and the Cayman Islands (2,017 CE) with magnitudes of 7.5 and 6.8, respectively. However, the comparison with the United States Geological Survey (USGS) dataset implies a relatively low potential for such seismic event layers on the basis of longer return periods of approximately magnitude-seven earthquakes (> 150 years) and only two potential matches within the historical record. Extrapolating the earthquake correlation over the entire core length, 7% of all recorded event beds can be theoretically attributed to seismic events. It is, however, questionable whether earthquakes follow such random patterns. The majority of the identified event layers derive, in accordance with the calibration of the historical record, likely from TC over-wash. The origin of six event layers remains unknown, but at least four of them occur in times of unreliable storm documentation without a clear TC track observation (< 1950 CE). These additional event layers could also originate from undocumented or inaccurately tracked TCs.

**Figure 3:**
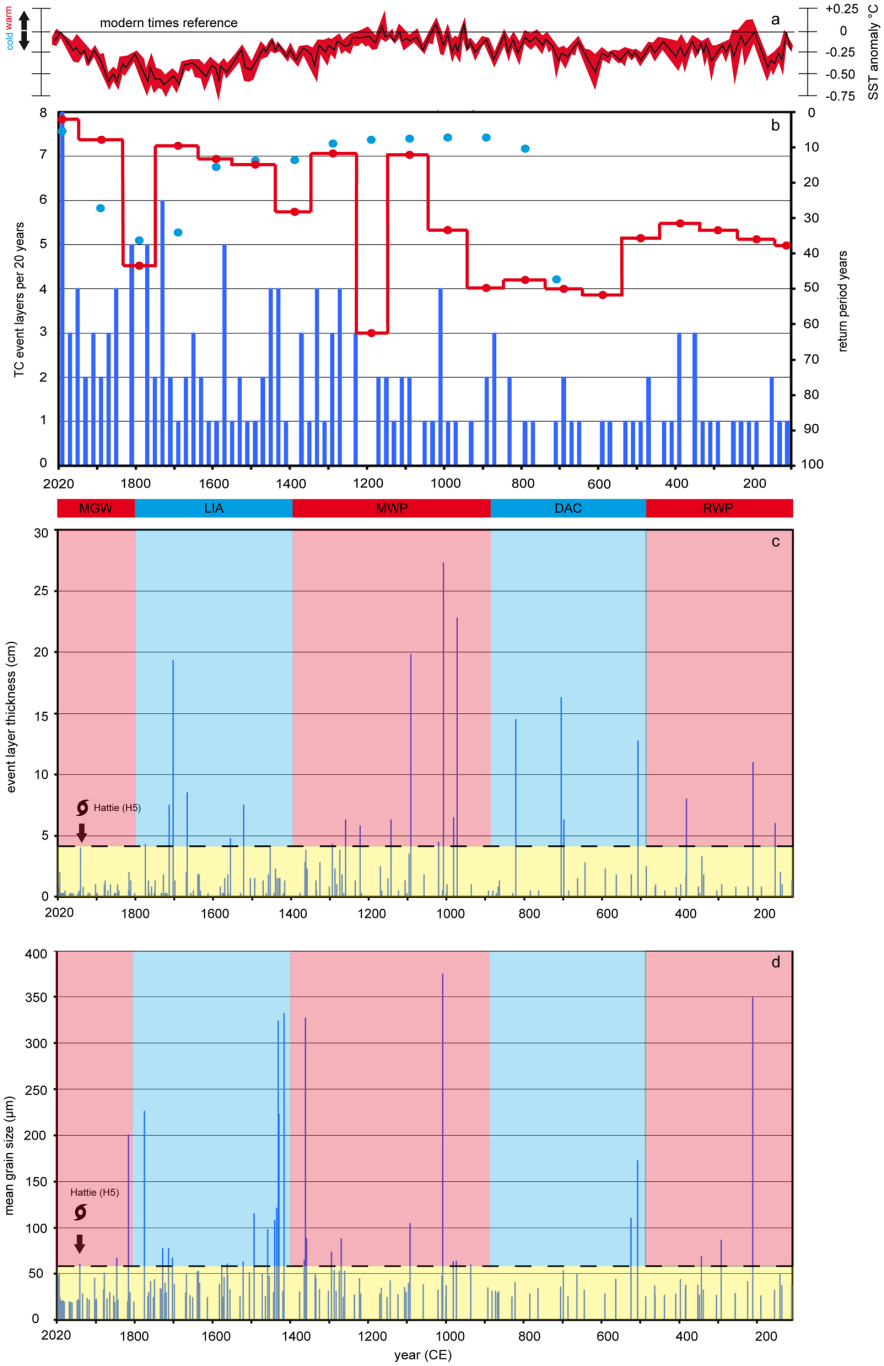
(**a**) The synthesis data of a global SST reconstruction^36^ are based on cumulated global^37^, Northern Hemisphere^38^ and western Pacific Warm Pool^39^ SST reconstructions. (**b**) Blue bars in histogram illustrate the total amount of all detected event layers per 20-year interval (n = 145). Red line is the 100-year average for TC return period as a quantitative proxy for TC frequency. The blue dots represent data from a previous study^16^. (**c**,**d**) Blue bars in histogram represent event-layer thickness (cm) and mean grain sizes (μm). Category 5 Hurricane Hattie (1961 CE) that passed over Lighthouse Reef was used as reference frame to determine textural outstanding event layers (yellow area).

The return period of TC events averaged over 100 years (T_100_)^16^ is a more quantitative indicator for TC frequency compared to the classical counting approach (activity = n_storm_/100) (Fig. 3b). We counted the number of event layers per 20-year period, calculated five individual 20-year event return periods T_20_ = 100/(n_event layers over 20 years_ × 5) and averaged the recurrence rates over one century (T_100_) to compare activity data with a previous Lighthouse Reef core^16^. The average frequency of TCs was relatively low (T_100_ = 35 +/− 3 years) during the Roman Warm Period (RWP) and seems rather constant from 100 to 500 CE. The Dark Ages Cold (DAC) is characterized from 500 to 900 CE by comparably constant but by even lower TC activity (T_100_ = 46 +/− 7 years). The evidence from both periods suggests a constant TC regime and low TC activity in the south-western Caribbean. The beginning of the Medieval Warm Period (MWP)—the most recent pre-industrial warm period (900–1,400 CE)—seems to be the beginning of a 200-year transition (900–1,100 CE) towards higher TC activity (1,100 CE-modern). This transition to a more active state (T_100_ = 12 years) ceases in the mid-MWP. The average MWP recurrence rates (T_100_ = 34 +/− 23 years) are somehow comparable to that of the RWP, but the standard deviation illustrates a very high variability ranging from minimum T_100_ = 12 (1,100–1,200 CE and 1,300–1,400 CE) to maximum T_100_ = 63 recurrence years. The maximum activity intervals are comparable with previous results indicating extremely high and constant TC activity (T_100_ = 4–7 years) during the MWP^16^. Obvious differences are, however, the higher variability and the slightly longer return periods in our record, which probably results in parts from the annual as compared to the near-annual resolution (2 years)^16^. A possible explanation for the significant drop between 1,200 and 1,300 CE could be the fact that event beds do not cover the bottom of the sinkhole entirely. Missing event layers within individual 20-year periods have a large influence on calculating the quantitative T_100_ average values, as seen impressively for this time interval. TC activity again decreases (T_100_ = 28 years) in the early Little Ice Age (1,400–1,500 CE) and then stabilizes (T_100_ = 9–15 years) from 1,500 to 1,700 CE during mid-end LIA. We observe the final drop in TC frequency back to the pre-transition level (T_100_ = 43 years) at the end of the LIA starting around 1,700 CE, which is 100 years later than previously described^16^. One possible solution for the temporal discrepancy could be problems of the previous study concerning the correlation between radiometric and varve counting data and related dating uncertainity^16^. The conspicuous 340-years age offset between both methods has been observed neither in the current BH6 core nor in the previous LOCO 1-core^28^. The weak linkage to an older carbon source^16^ is speculative and not supported by the other Great Blue Hole cores, which both indicate an extremely reliable core top dating as well as strong radiocarbon and varve age correlations. The decreasing trend in LIA TC activity obtained from core BZW-BH-SVC4^16^ seems to be similarly influenced by missed event layers around 1,720 CE, 1,840 CE and 1,900 CE. This argument may explain the differences regarding the onset and length of the decreasing period between this core and BH6. Nevertheless, there are still comparable sharp decreases of the TC return period in both cores at the end of the LIA followed by a 100-year (1,900–2,000 CE) increase in TC frequency (T_100_ = 8 years), which extends until the present day (T_100_ = 2 years > 2,000 CE). The average recurrence rate in the instrumental period is T_100_ = 5 +/− 4 years (core data) and indicates thus the highest frequency in the BH6 record. This high activity interval also corresponds very accurately to data obtained from the historical TC-strike record for Lighthouse Reef (T_100_ = 7 years). TC activity is generally higher in the second half (> 1,100 CE) than in the first half of the Common Era record, except for the short period from 1,700 to 1,900 CE. All these observations likely reflect a connection of TC activity with Common Era climate changes, which mainly control the underlying ocean-and atmosphere boundary conditions on longer time-scales. The comparison of quantitative T_100_ values between cores of the same study site and data from other regional TC archives should be treated very carefully due to local coring-site differences, grain-size proxy limitation and the uncertainty arising from randomly distributed event layer gaps or seismic events. The general trends in cores BH6 and BZW-BH-SVC4 are in any case altogether comparable and thus well suited to reflect the changes in long-term south-western Caribbean TC patterns.

**Figure 4:**
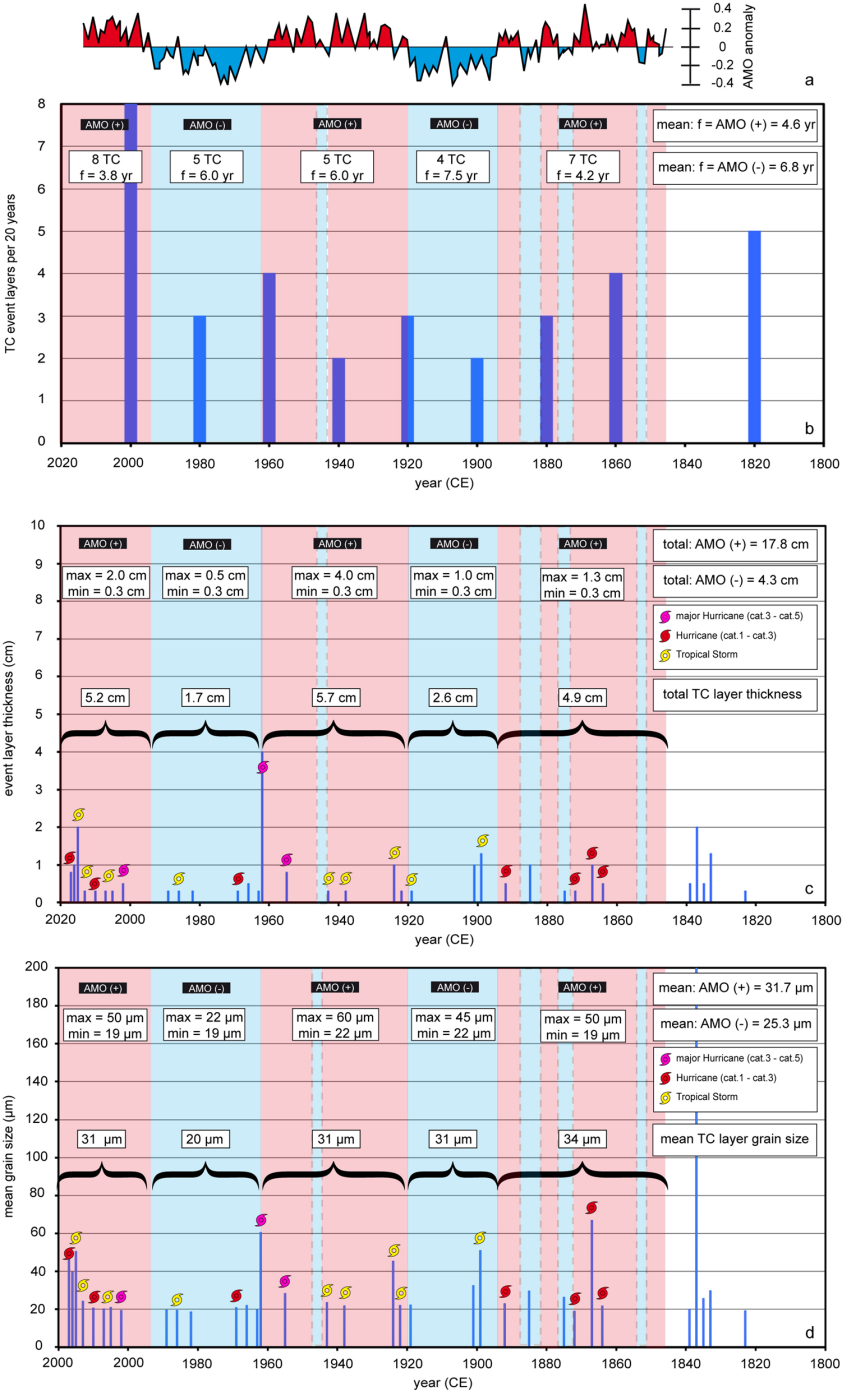
(**a**) 50–70 years AMO anomaly (red = positive phase; blue = negative phase) plotted on basis of NOAA/ESRL/PSD raw data (1,850–2,000 CE). (**b**) Calibration of TC activity with AMO phases of the instrumental record. (**c**,**d**) Calibration of event-layer thickness (total) and mean grain size (average) with AMO phases of the instrumental record.

A comparison of different historical record TC categories with event-layer thickness (Fig. 4c) and grain size (Fig. 4d) did not indicate significant correlations between cyclone intensity/duration and textural characteristics. Relatively large, but rather porous *Halimeda* chips are the major event layer constituents. Even weak tropical storms can easily mobilize such particles under low critical stress and initiate event layer formation^16^. Another factor contributing to the lack of correlation is the path and the energy of storm systems, which cannot be considered on longer time scales. Long reef recovery time (decades) and high TC activity intervals (T_100_ < 12 years) are other important factors controlling the availability of source sediment and thus the textural characteristic of event layers. The importance of reef recovery time is expressed by a weak but nevertheless statistically significant correlation (r = 0.22; p = 0.0057) between layer thickness and time between individual TC strikes. As a result of the complex interplay of factors controlling thickness and grain size of event layers, a reconstruction of TC intensity/duration is not possible so that this study focuses only on the temporal pattern of TC frequency.

It is, however, possible to observe some qualitative long-term patterns of textural data in the record (Fig. 3c,d). Average grain size of event layers appears to be finer from 100 to 1,100 CE, which is in particular illustrated by the occurrence of 10 outstanding event layers (referenced to the textural characteristics of Hurricane Hattie) in comparison with 20 outstanding event layers in the upper part of the record. Event-layer thickness on the contrary seems to be higher (6–27 cm) in the lower part of the record as compared with the upper core part (4–20 cm) with an equal number of outstanding event layers in both cases (n = 12). Taking the lower TC frequency during the RWP and DAC into account, the enhanced event layer thickness of basal core parts supposedly reflects the relationship of higher abundance of erodible reef material and larger time intervals between individual TC strikes. In return, the lower abundance of erodible reef material during times of higher TC activity likely explains the lower event-layer thickness in upper core parts. It is anyway very difficult to fully exclude the other effects controlling event-layer thickness or grain size as a consequence of, e.g., storm distance, duration or wave energy. Our long-term textural data interpretation is, therefore, far from being complete, especially for event layer grain sizes.

Five event layers of the Common Era record are of particular interest (1,113 CE, 1,029 CE, 944 CE, 843 CE and 726 CE). Two of these five TC strikes coincide with major drought phases in the Mayan Lowlands (820–870 CE and 1,020–1,100 CE). Several cm thick event layers coinciding with such extremely dry periods might reflect a variation in the strength of the Caribbean Low-Level Jet (CLLJ) in response to an increase in pressure gradients between the North Atlantic High and the Pacific ITCZ^40^. Such “dry-period event layers” may theoretically be attributable to large swells associated with a strengthening of the CLLJ. There is, however, no direct correlation of dry phases, CLLJ strength and TC event-layer occurrence verifiable for the historical records, which makes it rather speculative to consider this linkage during MWP and DAC times. In general, swells caused by CLLJ strength variations are furthermore not comparable in terms of energy and over-wash potential with several kilometres long storm surges and storm waves. The CLLJ linkage offers, therefore, not a convincing explanation for individual event layers coinciding with major drought phases. The same event layers could equally just be sporadic TCs that stalled over the site despite drought periods. The DAC and MWP droughts were previously interpreted as a crucial factor of the Classical Maya culture demise^41,42^. The other three emphasized TC strikes occur directly prior to, during and after the drought periods. It could be speculated that TC landfalls in the Maya Lowlands caused heavy destruction and flooding and might have contributed to the stress during drought phases further pushing the Maya civilization towards their limits.

The described long-term trends in TC activity precisely follow a Common Era global SST model^36^, based on cumulated global^37^, Northern Hemisphere^38^ and western Pacific Warm Pool^39^ reconstructions (Fig. 3a). The temperature curve indicates a strong connection of TC patterns with rising/high (active) and falling/low SSTs (inactive). Multi-decadal SST variations, which are of great importance to modulate the historical record TC activity, are not considered in such long-time comparisons. TC activity (Fig. 3b), event-layer thickness (Fig. 3c) and mean grain size (Fig. 3d) apparently underlie multi-decadal variations over the Common Era. We compared a regional SST reconstruction (1,750–2,017 CE)^43^ and AMO anomalies (Fig. 4a) with historical record TC frequency core data to highlight the importance of such multi-decadal SST and TC variations. Three positive (n_storm_ = 19) and two negative (n_storm_ = 9) AMO phases are correlated with high (> 2,000 CE, 1,920–1,960 CE, 1,850–1,890 CE) and low (1,960–2,000 CE, 1,890–1,920 CE) TC activity intervals and positive/negative SST anomalies, respectively. The average return period is shorter (T_20_ = 4.6 years) in positive AMO phases than during negative phases (T_20_ = 6.8 years; Fig. 4b). Similar results were obtained when comparing total event-layer thickness (Fig. 4c) and grain-size averages (Fig. 4d) with AMO phases. The total thickness and mean grain size of event layers in AMO + phases are clearly higher and coarser (17.8 cm, 31.7 μm), respectively than in negative ones (4.3 cm, 25.3 μm). Within the instrumental record, there is clear evidence for coarser/finer and thicker/thinner event layers during AMO+/AMO− phases, in which higher/lower SST prevails in the tropical Atlantic. Based on this AMO/SST calibration, we propose the use of event-layer characteristics (mean grain size/layer thickness) and TC counts for AMO signal detection.

**Figure 5:**
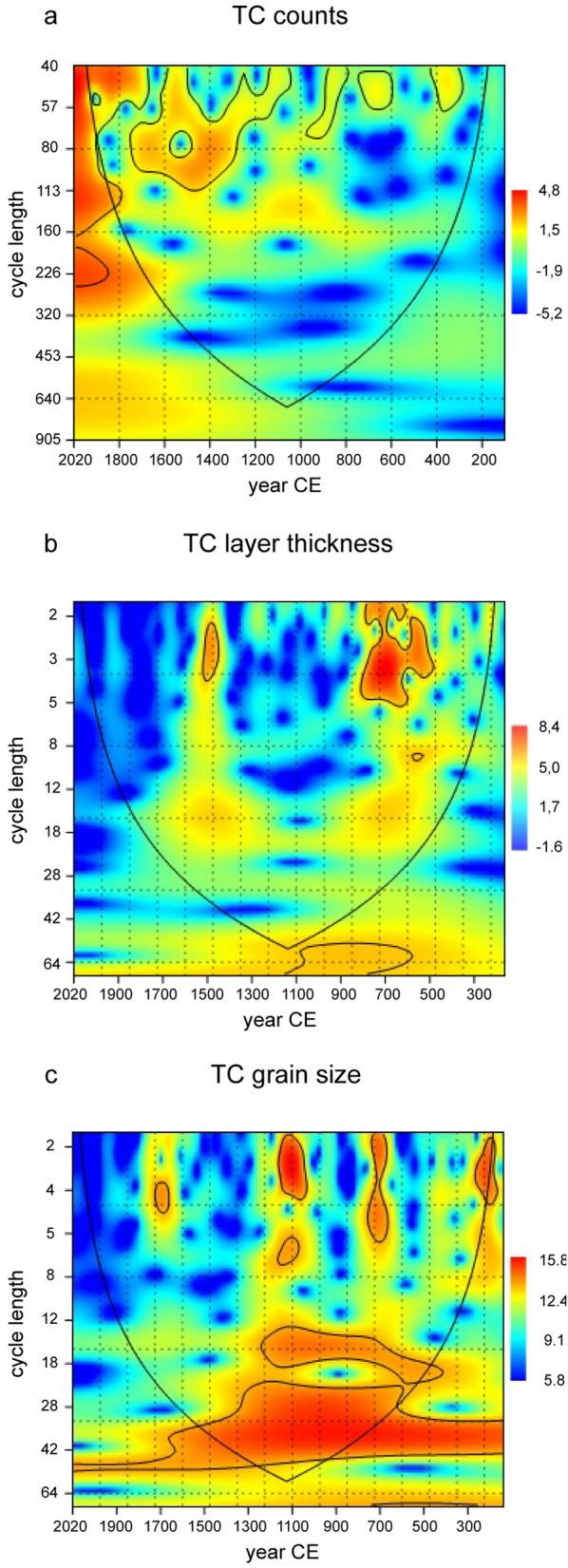
Wavelet power spectra comprising TC counts (**a**), TC layer thickness (**b**) and TC grain size (**c**). Statistically significant cycle lengths (p < 0.05) with confidence level 95% are contoured and coloured orange to red. (**a**) TC frequency is influenced statistically significant by AMO variation over the entire Common Era. The detected mean cycle length is around 44–64 years. (**b,c**) Both layer thickness and mean grain size are also linked to periodicities of 44–64 years.

Wavelet analyses of event layer counts (Fig. 5a), thickness (Fig. 5b) and grain size (Fig. 5c) data indicate a statistically significant cyclic signal over nearly the entire Common Era ranging from minimum 300–2,017 CE. The mean cycle length varies in a 95% probability interval between 44 and 64 years and is either compatible with the periodicity of the AMO signal (50–70 years) or multi-decadal variations of ENSO amplitudes^19^. The results from the wavelet analysis support the assumption, that secondary climate mode variations modulate the TC-activity not only during the Instrumental Era; they rather occur over the entire Common Era timeline. We suspect the AMO phase changes to be an appropriate driver for the secondary (multi-decadal) TC activity variation, because of a strong historical record correlation of AMO indices/anomalies with TC counts, textural parameters and tropical Atlantic SST variations (Fig. 4a–d)^43^. The lack of intra-decadal (5–8 years) ENSO signals^19^ is another indirect argument for the proposed AMO control. Intra-decadal ENSO variations are closely linked to the multi-decadal ENSO amplitude variation. If the observed multi-decadal signals were really attributed to ENSO amplitude variations, intra-decadal signals would have been detected also in the wavelet power spectra. For these reasons, the AMO influence appears to be an important long-term mechanism for understanding past TC activity patterns. The secondary (multi-decadal) AMO control likely works in a subsequent and supportive manner with regard to the primary (centennial/millennial-scaled) long-term AMO mode forcing in response to Common Era climate variation^44^.

### Comparison with other TC records

The record of core BH6 coincides quite well with previous ones from the same location^16,28^ in an overlapping time window (800–2,017 CE). All cores from Lighthouse Reef indicate the highest TC activity during the MGW (1,900–2,017 CE), a distinct decrease (medium activity) over the LIA (1,400–1,900 CE) and a high activity since the mid-MWP (1,100 CE). The BH6 record adds an unprecedented period of 700 years of low TC activity that is attributable to the DAC (500–900 CE) and RWP (100–500 CE). The dating of rough trends and the calculated values of TC return periods, however, differ among the three Lighthouse Reef cores. Despite a general coincidence of broad TC activity patterns, there are some limitations in the previous studies. The previously used approaches to identify TCs are certainly less accurate because they are largely based on visual colour differences, quantitative event-layer thickness measurements and semi-quantitative grain-size estimations. The additional consideration of event-layer geometry, fine material abundance, quantitative background and event-layer grain-size measurements, a historical record grain-size calibration and innovative XRF scanning, as presented here, is an important step forward that ensures a more reliable TC layer identification. The two cores from the previous studies are also significantly shorter and exhibit only a 2–4 years resolution as compared to the annual resolution of the BH6 core. These methodological differences likely explain the observed variations between the calculated TC return periods. Another noteworthy limitation of one of the previous studies is the appearance of age dating problems and the occurrence of a 340 years age offset between varve and radiocarbon dating within the BZE-BH-SVC4^16^ core. This age offset is likely to large parts responsible for the observed difference in dating of rough Common Era TC activity trends, as exemplified in the late LIA drop discussion. The continuous offshore atoll records, characterized by annual to multi-annual resolution, are unfortunately not comparable with some discontinuous and decadal-resolution records obtained from coastal lagoons^20^ and marshes^22,23^ of the Belize mainland coast. These records indicate a higher TC activity during the LIA and RWP (100–500 CE) and lower TC activity within the MGW and MWP (900–1,400 CE). The significance of these discontinuous TC activity reconstructions remains strongly restricted, because of an exceptionally low event-layer preservation potential, which is caused by erosional unconformities and other geomorphological processes related to changes of accommodation space, sediment supply and subsidence.

A continuous coastal lagoon record from Puerto Rico^45^ with decadal resolution demonstrates a completely different behaviour of long-term TC patterns than the Belize offshore atolls, i.e. high activity during the MGW, the DAC and the RWP and low activity during the LIA and the MWP. The general difference to the Puerto Rico record can be best explained by the occurrence of four major Caribbean TC clusters (Suppl. 3a)^46^. Landfalls over Puerto Rico (north-eastern Caribbean) derive largely from a combination of cluster 3 and 4 TC tracks, which originate in the Western, Central and Eastern Atlantic (Suppl. 3d,e). Landfalls over Belize are characterized by a general mixture of TC tracks belonging to clusters 2 and 4 (Suppl. 3c,e). TC landfalls on the Yucatan Peninsula belonging to cluster 2 have due to their genesis region (Western Caribbean) no impact on sedimentary records obtained from Puerto Rico. TC tracks of cluster 3 are generally not attributable to Belizean archives. Their major track direction towards the north-east originating from the genesis region in the Central-Eastern Atlantic favours landfalls in the north-eastern Caribbean region and along the North American east coast. The discrepancy of the Common Era TC patterns between Belize and Puerto Rico records are best explained by these major differences of clusters 2 and 3. This explanation is sound despite a possible overlap of cluster 4 TCs. Cluster 4 consists of two sub-clusters^46^ reflecting two major genesis regions of the same latitude, but different longitude (Caribbean Sea and Western Atlantic). TCs originating from these sub-clusters could exercise for one thing independent and different impacts on both sites considering the ordinarily given north-east-ward cyclone path.

Another continuous and low-resolution cyclone record from the northern Antilles (St. Martin^47^) indicates, however, a TC activity pattern, which is more comparable to the one of the south-western Caribbean. A rough accordance with the Belizean TC activity pattern is on the other hand also conceivable. Cluster 4 TCs originating from the Western Atlantic sub-cluster (genesis region east of the northern Antilles) may well migrate under appropriate atmospheric conditions initially westwards and cross the northern Antilles. The same TCs may secondarily move over the Yucatan Peninsula following the north-eastern migration path. The bipartite nature of cluster 4 is an admittedly weak, but satisfactory explanation for the observed general differences and similarities between our south-western and two north-eastern Caribbean records.

Two North American records (Massachusetts^24^ and Florida^25,48^) are influenced due to their spatial distribution by practically all TC clusters, which makes a comparison really challenging. However, they all differ in general from the Belizean TC records. A common similarity between the individual annually to decadally-resolved North American archives is the low TC activity over the MGW and LIA, a higher TC activity within the MWP and DAC as well as a highly site-specific TC variability during the RWP. The continuous and multi-decadal^1,26^ to annually^27^ resolved Bahamas records are relatively similar to the North American archives with low TC activity during the MGW/LIA and high TC activity during the MWP/DAC.

TC activity patterns strongly depend on the different TC cluster, which cause in particular large local activity variations. This limitation makes a quantitative comparison and hazard assessments at basin-scale very difficult. A common feature seems to be, however, the occurrence of a general TC activity shift in the wake of the MWP climate change. All north-western Caribbean and North American records change from a more active TC regime (< 1,100 CE) towards a more inactive state (> 1,400 CE). The south-western Caribbean records show a remarkably similar temporal, but reversed transition from a less active (< 1,100 CE) to a more active state (> 1,400 CE). This latitudinal pattern can be explained by changes in the position of the ITCZ as the potential driver. The ITCZ shifted in the wake of this climate change from a relatively northern position (0–1,000 CE) towards a more southern location (1,000–1,400 CE)^1^. This southward ITCZ migration enhanced the potential for TC landfalls over the wider Yucatan Peninsula, which concurrently suppressed the landfall potential in the Northern Caribbean and along the North American east coast (latitudinal shift of genesis regions).

### Common Era TC activity: a conceptual and comparative view

**Figure 6:**
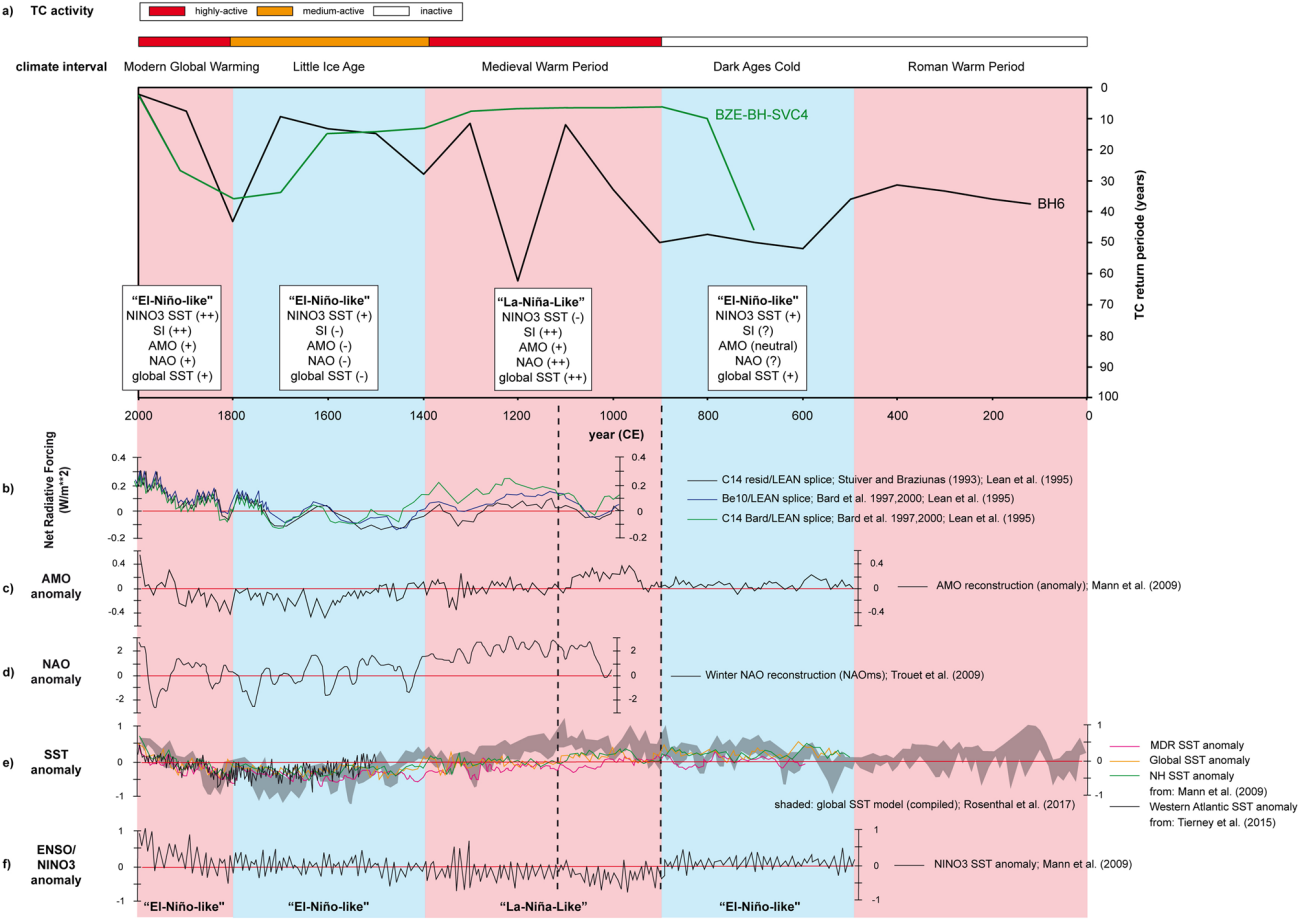
(**a**) Comparison of TC activity obtained from the BH6 (black line) and BZE-BH-SVC4^16^ (green line) records in the wake of Common Era climate changes (red = warm interval; blue = cold interval). (**b**–**f**) Climate phenomena and SST responses on common Era climate changes triggered by a combination of solar irradiance^53–56^ and volcanism^50,57,58^. (**b**), involving AMO^51^ (**c**), NAO^52^ (**d**), individual global^51^, Northern Hemisphere^51^, Western Atlantic^43^ and MDR^51^ SST curves, a global synthesised Common Era SST model^37^ (**e**) as well as ENSO/NINO3 reconstruction^51^ (**f**). The dashed lines roughly frame the transition interval of the changing Common Era TC activity in succession of a 200-year hydro-climate variation in response to the onset of the MWP climate anomaly.

The annually-resolved Great Blue Hole TC record offers the great potential to review the connection of long-term TC activity patterns and Common Era climate variability. We provide, largely based on qualitative observations, a conceptual and comparative view of all involved tropospheric and oceanographic processes, which have likely shaped the TC activity patterns of the south-western Caribbean in response to Common Era climate variation (Fig. 6). Variations in solar irradiance (Fig. 6b) have been previously considered as a main external driver for late Holocene climate changes^44^. Volcanic forcing is another potential and closely linked natural driver of the Common Era climate variability as it may alter solar insolation. High volcanic forcing activity intervals induce enhanced sulfate aerosol injections into the stratosphere, which shield the earth surface from incoming solar radiation and consequently cause regional and global cooling tendencies^49^. The volcanic forcing effect on air- and surface ocean-temperature variability is, however, considering the past 2,500 years largely realised on inter-annual-to-decadal time scales^50^. Both climate driver mechanisms have in common that they cause subsequent feedbacks of important climate phenomena. Therefore, the climate phenomena of the ENSO^51^ and AMO^51^ as well as to some minor extent the NAO^52^ have been considered in our conceptual view as potential internal TC-controlling mechanisms, because they have likely modulated the boundary conditions for TC formation by exercising primary control on basin-scaled SST patterns (Fig. 6e) and vertical wind shear stress dynamics. There is, however, also a relevant secondary/multi-decadal AMO control apparent, which needs to be considered. We qualitatively compared relative long-term reconstructions of these climate modes with our TC activity reconstruction, in order to decipher possible key control mechanisms, and independently discussed four of the five Common Era climate intervals in this regard. The considered climate mode and SST reconstructions reach not far enough back in time to make the RWP subject of the discussion (Fig. 6).

A causative explanation for the onset of the DAC could be a temporal overlap with a phase of strong tropical and high-latitude volcanic activity (500–600 CE)^50^. The related relatively high global aerosol injection would certainly have exercised control on regional and global SST patterns via attenuation of solar insolation. The compiled global SST curve^36^ supports this argument by indicating a global negative SST anomaly during the sixth century^50^. The reconstructed decrease of the south-western Caribbean TC recurrence rates (T_100_ = 46 years) relative to the RWP (T_100_ = 35 sears) is probably attributable to this SST anomaly, hampering a frequent TC formation in the early DAC. However, the temporally limited effect of volcanic forcing and its feedback on SST is not sufficient to explain the persistent 400-year trend of low TC activity. The continuation of the low activity trend may, however, satisfactorily explained by considering persistent “El-Niño-like” conditions in the mid and late DAC (Fig. 6f). Such ENSO conditions hamper the TC formation potential as a result of strong vertical wind shear stress, stronger trade winds and an overall greater atmospheric stability^17^. Stable “El-Niño-like” conditions should concomitantly induce a regional positive SST anomaly in the tropical western Atlantic^59^. The MDR SST reconstruction^51^ provides some evidence for slightly enhanced tropical Atlantic SST during the DAC, which would theoretically favour a more frequent TC formation. The results of a previous Great Blue Hole study^16^ indicate, however, that basin-scaled oceanographic variables and climate modes seem to be more important than local MDR SST conditions, with regard to TC-activity modulation. This argument in turn explains the weak effect of positive MDR SST on DAC TC-activity. A good explanation for the low internal variability, especially on multi-decadal time scales, may be the coincidence with a long-term stable and largely neutral AMO phase. For the outlined reasons, the early DAC drop of TC activity and the continuation of the low TC activity regime are best explained by a volcanically initiated global cooling and tropospheric ENSO conditions, which hampered frequent TC formation.

The onset of the MWP (~ 900 CE) may be associated with a phase of increased solar activity^44^ and reduced tropical and high-latitude volcanic activity^50^. Such preconditions could have been a potential trigger of extensive hydro-climate changes during the transition into the MWP climate anomaly. These changes in hydro-climate include a global ocean heating, as indicated by the outstanding positive SST anomaly in the compiled SST curve^36^, following high rates of radiative solar forcing (Fig. 6b). A heating of the extra-tropical Pacific Ocean especially favours the development of long-term stable “La-Niña-like” conditions^60^ (Fig. 6f), which should involve theoretically a negative western Atlantic SST anomaly^59^. The MDR SST reconstruction^51^ supports the assumed “La-Niña” conditions effect on regional SST by indicating slightly decreasing tropical Atlantic temperatures. The reorganisation of the ENSO dynamics contains additionally important modifications in the North Atlantic atmosphere circulation such as lower vertical wind shear stress conditions, a strengthening of westerly winds and a weakening of the trade-wind system^17^. These atmospheric conditions would have favoured in general, and in combination with a positive basin-wide to globally-scaled SST anomaly, the development of a higher TC activity regime during the MWP. Long-term positive AMO (Fig. 6c) and NAO (Fig. 6d) modes would coevally contribute to develop suitable ocean and atmosphere conditions for a centennial-scaled enhanced TC activity and for a multi-decadal-scaled higher variability of TC activity, respectively.

The LIA climate change is the result of lower solar irradiance^44^, two distinct phases of enhanced global volcanic activity or a combination of both, which caused global air- and sea surface temperature cooling, in particular at the beginning and at the end of the LIA^57^. The onset of the LIA cooling (1,300–1,400 CE) coincides with the most active volcanic half-century of the past millenium^58^. Repeated explosive volcanism on its own may act as a trigger of climate change, independent of the precondition that the orbital configuration of the earth favours low solar insolation. The LIA climate change, likely initiated by multi-decadally paced explosive volcanism and maintained by ocean and climate mode feedbacks^58^, did therefore not mandatorily require a solar trigger. The reconstructed TC activity pattern of the LIA is characterised by a TC activity decrease (1,300–1,450 CE), a stabilisation back to MWP conditions (1,450–1,700 CE) and a considerable drop back to very low TC activity after 1,700 CE. These rough trends accurately follow the two supposedly volcanically-induced cooling intervals^57,58^ and the climate-mode responses to the LIA climate change. Global^36^, northern hemisphere^37^, MDR^51^ and western tropical Atlantic^43^ SST reconstructions collectively indicate decreasing regional and global SST, however, with the specification of a step-wise, two-phase decline in the early and late LIA and an intercalated short warm interval. The gradual reorganisation of the ENSO dynamics towards “El-Niño-like" conditions and the persistent negative AMO and NAO modes, following a volcanically initiated climate variation^58^, apparently sustained the observed LIA TC-activity pattern as a combined result of inappropriate atmosphere and ocean conditions.

The MGW climate change (> 1,900 CE) is either the result of naturally increasing solar irradiance (Fig. 6b) or caused by anthropogenic forcing, even though the second explanation is still a major point of the recent climate change debate. The high TC activity of the MGW climate interval is, however, in either case attributable to ocean heating processes, as indicated by an obvious temperature rise in all considered SST curves (Fig. 6e) and climate-mode responses. Long-term stable “El-Niño-like" conditions should, however, hamper the TC formation potential from an atmospheric point of view. This disagreement is apparent but may be best explained by the amplification of the positive AMO mode that causes a relative weakening of local ENSO effects^59^. The discrepancy is, however, probably simply the result of an increasing significance of anthropogenic forcing, which originates from rising atmospheric carbon dioxide concentrations.

Our Common Era TC-activity study provides four key results: (1) The TC-activity of the south-western Caribbean generally shifted from a less active (100–900 CE) to a more active state (900 CE—modern). (2) We identified continuous and at least 1,700-year-long multi-decadal signals of 44–64 years periodicity. These cyclic signals have been interpreted as an indicator for a multi-decadal AMO modulation of Atlantic TC-activity, which encompasses nearly the entire Common Era. (3) Climate-mode responses to external forcing mechanisms (solar irradiance and volcanism) have likely operated over the Common Era as the internal control mechanism for long-term TC activity. (4) The conceptual consideration of Common Era climate variability and climate-mode responses indicates a higher importance of basin-scaled ocean and atmosphere dynamics than variations in regional MDR boundary condition for shaping the long-term TC activity patterns.

## Methods

### Fieldwork in Lighthouse Reef

Seven sediment cores were collected from the bottom of the Great Blue Hole of Lighthouse Reef, Belize (17° 86′ N, 87° 32′ W), along a W-E transect from a small boat using a portable Rossfelder P3 electrical vibrocore system and 6 m long aluminium tubes, equipped with copper core catchers. In order to extend core length to 9 m, two aluminium pipes were glued together using 50-cm-long aluminium sleeves. Maximum core length ranges from 1.80 m (shortest core BH1) to 7.55 m (longest cores BH6 and BH7). The coordinates at the coring sites were registered using a GPS instrument. After retrieval, cores were cut into 1.5 m-long sections, transported by air cargo from Belize City to Frankfurt am Main, Germany, and stored in a cold room at 4 °C.

### Core description: photo documentation and stratigraphy

In the core laboratory, all tubes of BH1, BH6 and BH7 were opened and cut in halves lengthwise using an angle grinder and an ultra-thin copper sheet (0.7 mm). Photo documentations of the shortest core BH1 (17° 18.943′ N, 87° 32.071′ W) and the longest cores BH6 (17° 18.957′ N, 87° 32.098′ W) and BH7 (17° 18.950′ N, 87° 32.118′ W), the latter two from the approximate centre of the Great Blue Hole, were carried out at the University of Bern using a Geotek-MSCL-S (Multi-Sensor Core Logger). Based on length and preservation, core BH6 was selected for sampling. During the coring process, the core top of BH6 had been lost due to liquefaction. Therefore, cores BH6 and BH7 were spliced together by means of prominent event layers to replace missing material. This led to a maximum core length of 8.55 m and achieves the highest possible temporal resolution from modern times throughout almost the entire Common Era. By using supportive high-resolution core pictures (Fig. 1e), visual core description was done, including descriptions of different unconsolidated lithologies, measurements of mean annual layer thickness, differentiation of background versus event deposits, description of sedimentary structures, registration of macrofossils and characterization of sedimentary contacts (Suppl. 4).

### Systematic sampling

Background sedimentation was characterized previously by an annual sedimentation rate of 2.5 mm per year^28^. Following the given sedimentation rate of constant background deposition and visual identification of annual layers, approximately 3,000 samples (5 g bulk material for quantitative textural analysis) and subsamples (400 mg bulk material for stable isotope analysis) were taken consequently and systematically over the entire spliced 8.55 m long core at 2.5 mm intervals (Suppl. 5).

### Age dating: varve counting and radiocarbon calibration

To gain an age model for event-layer timing, varves were counted visually in the core sections and supportive by high-resolution core photos on basis of colour differences and measurements of laminae thickness. The mean sedimentation rate in BH6 core amounts to 2.55 +/− 0.05 mm/year confirming the results of earlier studies. Within deeper core sections, lamination is sometimes blurry and not as easy to identify as in younger core sections resulting in estimated varve counting errors of 5%. The age to depth correlation was made on the basis of the varve counts and background sedimentation rates (Suppl. 6). As an independent age determination, five bulk samples enriched in entirely marine organic matter were selected from varved core sections at 5.75 cm, 87.25 cm, 336.50 cm, 586.50 cm and 852.00 cm core depth for ^14^C AMS geochronology (Suppl. 7). For our samples, Beta Analytic Inc., Miami, Florida provides average δ^13^C values of − 16.0‰. Organic residue was separated from bulk sediment by dissolving carbonate material with HCl, washing the sample with NaOH, and repeating until no carbonate material remained. The organic matter dating^61^ has been undertaken by Beta Analytic Inc., Miami, Florida with Accelerator Mass Spectrometry (AMS) in order to ensure the highest level of possible age-dating quality. The conventional radiocarbon ages from marine organic residue were converted to calibrated years before present using BetaCal3.21 MARINE13 calibration curves (High Probability Density Range Method)^62^. All calibrated ages are presented with a 2-σ error with 95.4% probability. The ΔR factors used in reservoir correction of marine carbonate sediment age are Delta-R =  − 23 ± 26 from nearby Glovers Reef Atoll, as the best available site-specific ΔR value for the Great Blue Hole and global marine reservoir age of 405 years. A further calibration step (Fig. 2) was undertaken by regression lines between varve age (year CE), calibrated radiocarbon age (year CE) and core depth (cm), allowing the development of a consistent and robust anchored age-depth framework with determination coefficients of r^2^ = 0.98 (^14^C) and r^2^ = 0.99 (varves). Towards the lowermost core section, an increasing age offset between both age determination methods occurs. The uncertainties between layer counting and AMS dating in deeper core sections represent a minor data limitation. Independent ^14^C age determination helps not only to constrain the absolute timing of TC events over the entire BH6 sediment core, but also to document that there are no gaps at the core top or within core sections. There appears to be no hiatus at the top of the sedimentary record of BH6 core as evidenced by (1) a very thick and coarse-grained event layer at 21 cm core depth correlated with great certainty to category 5-hurricane Hattie that struck Lighthouse Reef in 1961 CE^63^, and (2) a ^14^C radiocarbon age near the core top (5.75 cm) indicating post “Bomb Effect” conditions.

### Quantitative textural analysis and sediment characterization

Textural data that have previously been obtained in much lower resolution and by visual estimation only^16,28^_,_ now have been collected in a quantitative way and analysed comprehensively over the entire core length at annual resolution level. For textural analyses, a total of 2,915 samples with 2.5 mm sampling intervals, ensuring sufficient material (5 g) for all necessary processing steps, have been first washed and sieved through standard grain-size sieves of 2 mm, 1 mm, 500 μm, 250 μm, 125 μm and 63 μm. Two recent studies^64,65^ have demonstrated, that the fine fraction (< 63 μm) alone may be sufficient to identify event beds in reefal lagoon settings. To guarantee reliable quantitative grain size and sorting measurements, a more detailed analysis of fine material < 63 μm was performed by using a laser-optical particle analyser (HORIBA Laser Particle Analyser-950), which runs with 2 g fine material (< 63 μm) suspended in 0,4 N Na_4_P_2_O_7_ and demineralized water (Suppl. 8). Sediments were categorized by determination of classical sedimentary parameters (mean grain size, sorting, skewness and kurtosis), following the “Geometric and Logarithmic Folk & Ward method” using the software package *gradistat*^66^. Poorly (1–2 Φ) to very poorly sorted (2–4 Φ), medium to coarse silt-sized couplets with absolute grain sizes ranging from 12 to 24 μm and layer thicknesses < 2.5 mm represent the annual background sedimentation of biogenic fine material. The mean grain size of the background sediments is 20 µm +/− 4 µm. Both poorly (1–2 Φ) to very poorly (2–4 Φ) sorted TC event layer units are several mm (> 0.3 mm) to 28 cm thick, depending on sediment source, cyclone strength, cyclone-track direction, proximity to sample site and residence time. Mean grain sizes of different event-layer lithologies range from 20–30 μm (coarse silt), 31–61 μm (very coarse silt), 62–121 μm (very fine sand), 133–226 μm (fine sand) and 263–374 μm (medium sand).

### X-ray fluorescence analysis: a new TC indicator

The other intact core half has been completely scanned at 0.3 mm intervals using 20 s integration times with an X-ray fluorescence core scanner (XRF; COX-ITRAX equipped with a Cr-anode-X-ray tube set to 30 kV and 40 mA) at the University of Bern, in order to implement a new and innovative high-resolution method for TC detection in calcitic sinkhole environments. With more than 20,600 measuring points and a sampling interval of 0.3 mm, XRF scanning can be a further high-resolution method for TC detection (Suppl. 9). Special attention was given in this context to peaks of the element ratio of Sr/Ca. Strontium is typically enriched in predominantly aragonitic marginal reef carbonate material, e.g., coral skeletons and *Halimeda* chips and depleted in lagoon sediments typically dominated by mollusk shells and foraminiferal tests^67^. Lagoonal sediment is the most likely source of background sediments in the Great Blue Hole, whereas coarser-grained sedimentary particles from the reef margin with high Sr amounts will potentially be transported into the sinkhole sediment trap and deposited within event layers.

### TC-identification

We used, in contrast to the previous studies, a multi-proxy-approach to ensure a more reliable and quantitative TC identification. Our approach requires at best a match with all five different event layer identification criteria: (1) grain-size (> 20–24 μm); (2) amount of fine material < 63 μm (< 85%); (3) layer-thickness (> 2.5 mm); (4) colour (yellow/brown) and (5) composition (Sr/Ca-ratio > 0.025). Precondition for a successful TC identification is a match with various criteria, because mean grain size is difficult to use as single identification tool due to several limitations (e.g. internal variations, layer geometry, coring site, core splicing and hydro-dynamical particle properties).

### Statistical wavelet analysis

TC counts, event layer thickness and mean grain size raw data (Suppl. 10) were used for statistical wavelet analyses (software package PAST version 3.14)^68^. The full dataset was inspected at various scales simultaneously, detecting periodicities at different wavelength within the 2000-year time series. The square correlation strength with the mother wavelet (“Morlet” with wave number 6) is shown on a coloured scale. The underlying algorithm is based on fast convolution of signals with the Morlet wavelet using a “Fourier Transformation”. Statistical significance level corresponding to p = 0.05 is plotted as a contour line (chi-squared test).

## Supplementary information

Supplementary Figures and Legends.

Supplementary Information 2.

Supplementary Information 3.

Supplementary Information 4.

Supplementary Information 5.

Supplementary Information 6.

Supplementary Information 7.

Supplementary Information 8.

## Data Availability

All data generated or analysed during this study are included in this published article (and its supplementary information files).
