# Supplementary material for: Caribbean cyclone activity: an annually-resolved Common Era record
Source: Sci Rep. 2020 Jul 16;10:11780. doi: 10.1038/s41598-020-68633-8 (PMC7367345; doi:10.1038/s41598-020-68633-8)
Supplement: Supplementary file 1 — Supplementary Figures and Legends. [file 41598_2020_68633_MOESM1_ESM.pdf]

Caribbean cyclone activity – an annually-resolved Common Era record

* Dominik Schmitt, Eberhard Gischler, Flavio S. Anselmetti, Hendrik Vogel

Suppl.-figures and Suppl.-tables

Suppl. 1: Seven core log files in chronological order (BH7/1, BH6/1, BH6/2, BH6/3, BH6/4, BH6/5, BH6/6). The multi-proxy-approach of TC identification was conducted on the basis of textural data (grain size fraction <63 µm, layer thickness and mean grain size), Sr/Ca ratios and sediment colour.

TC event layer identification: core log BH 7/1

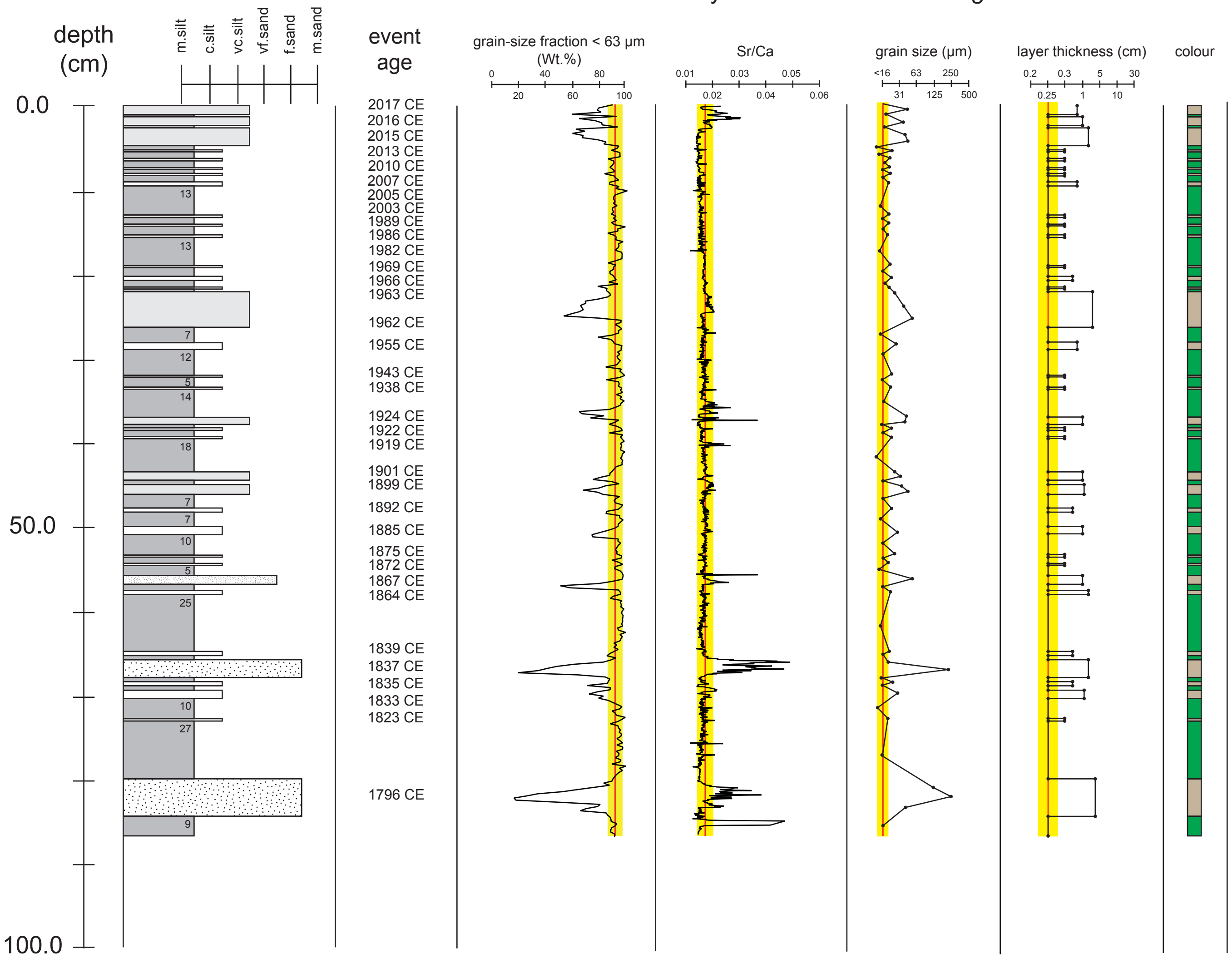

TC event layer identification: core log BH 6/1

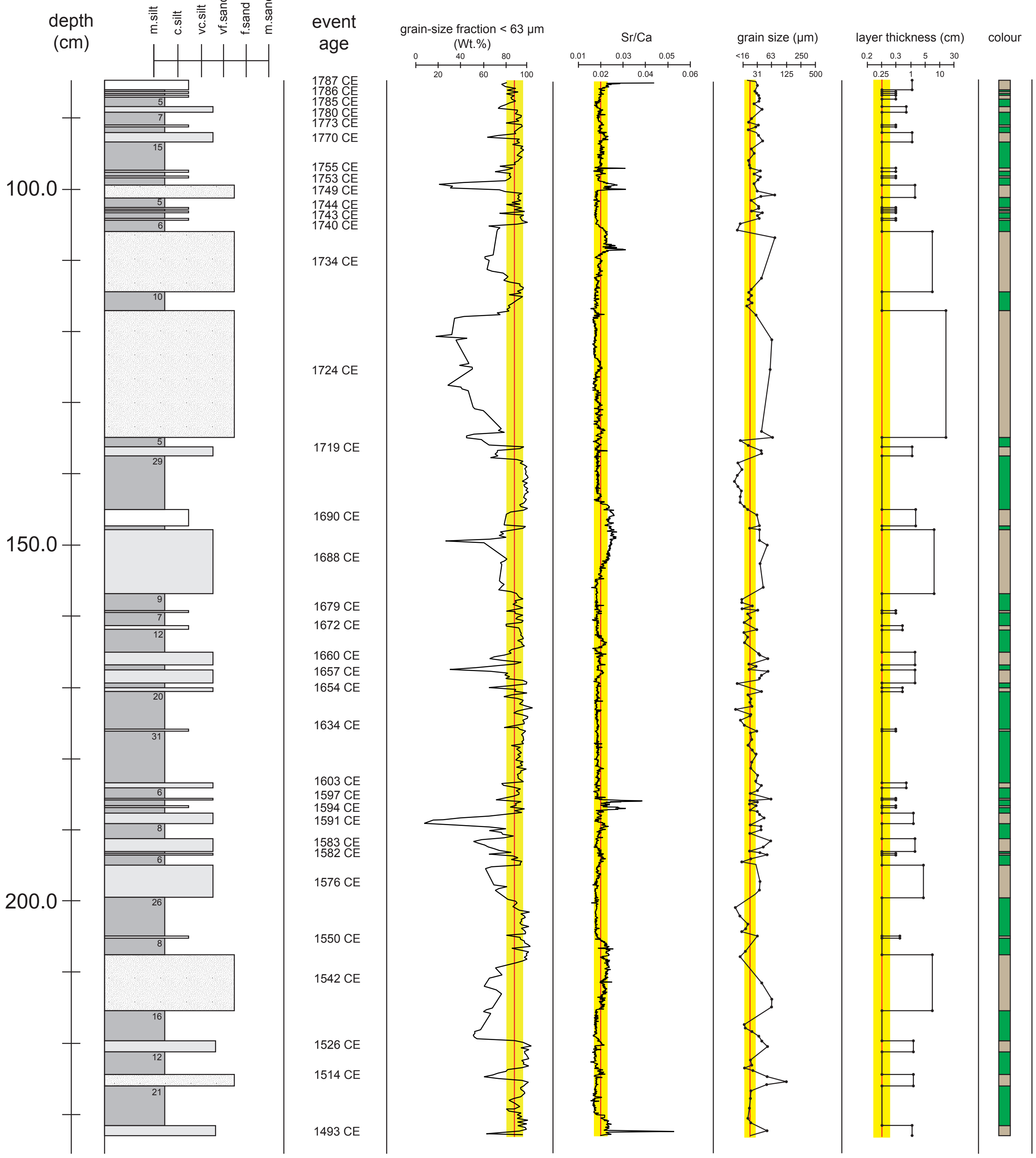

TC event layer identification: core log BH 6/2

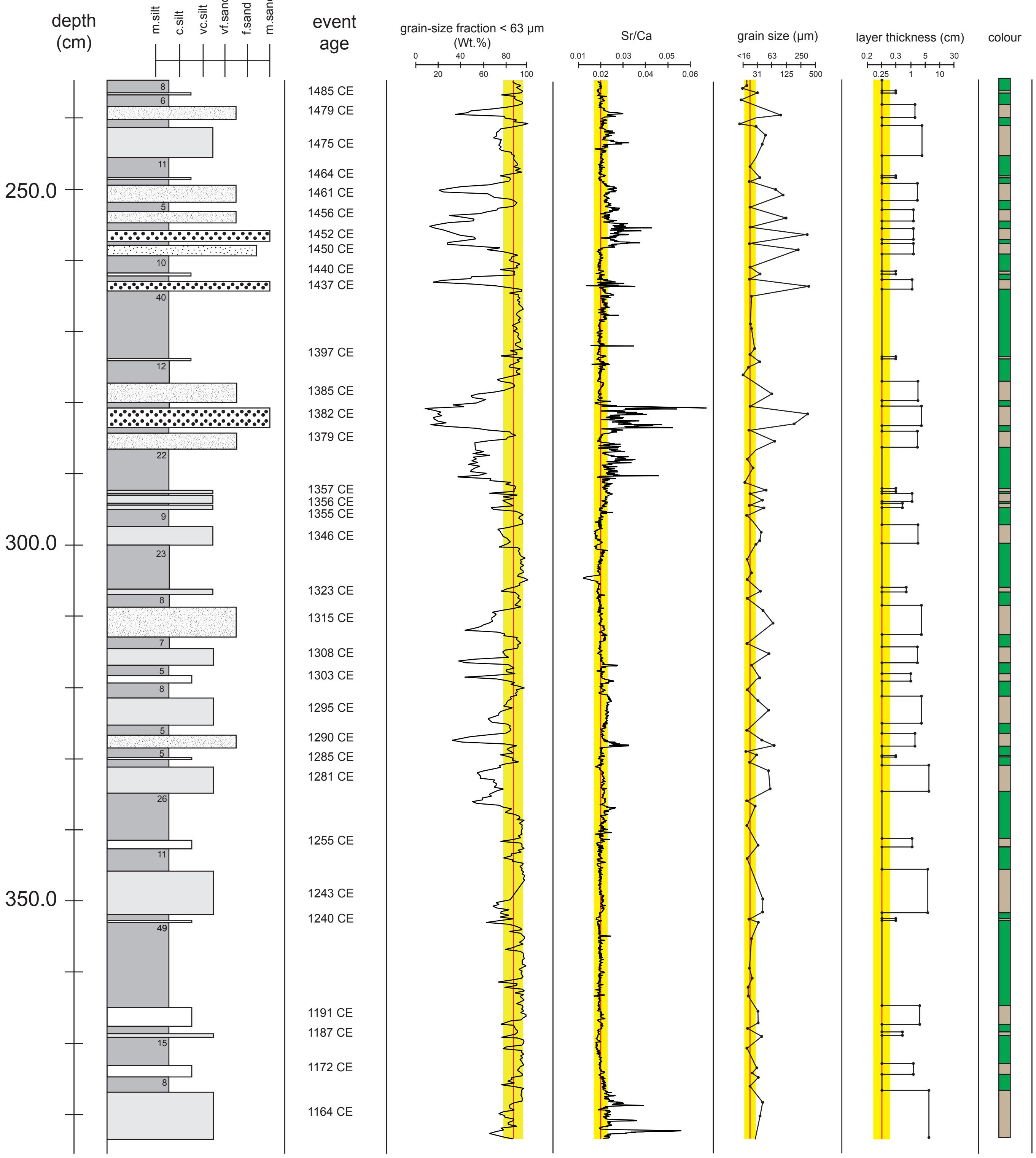

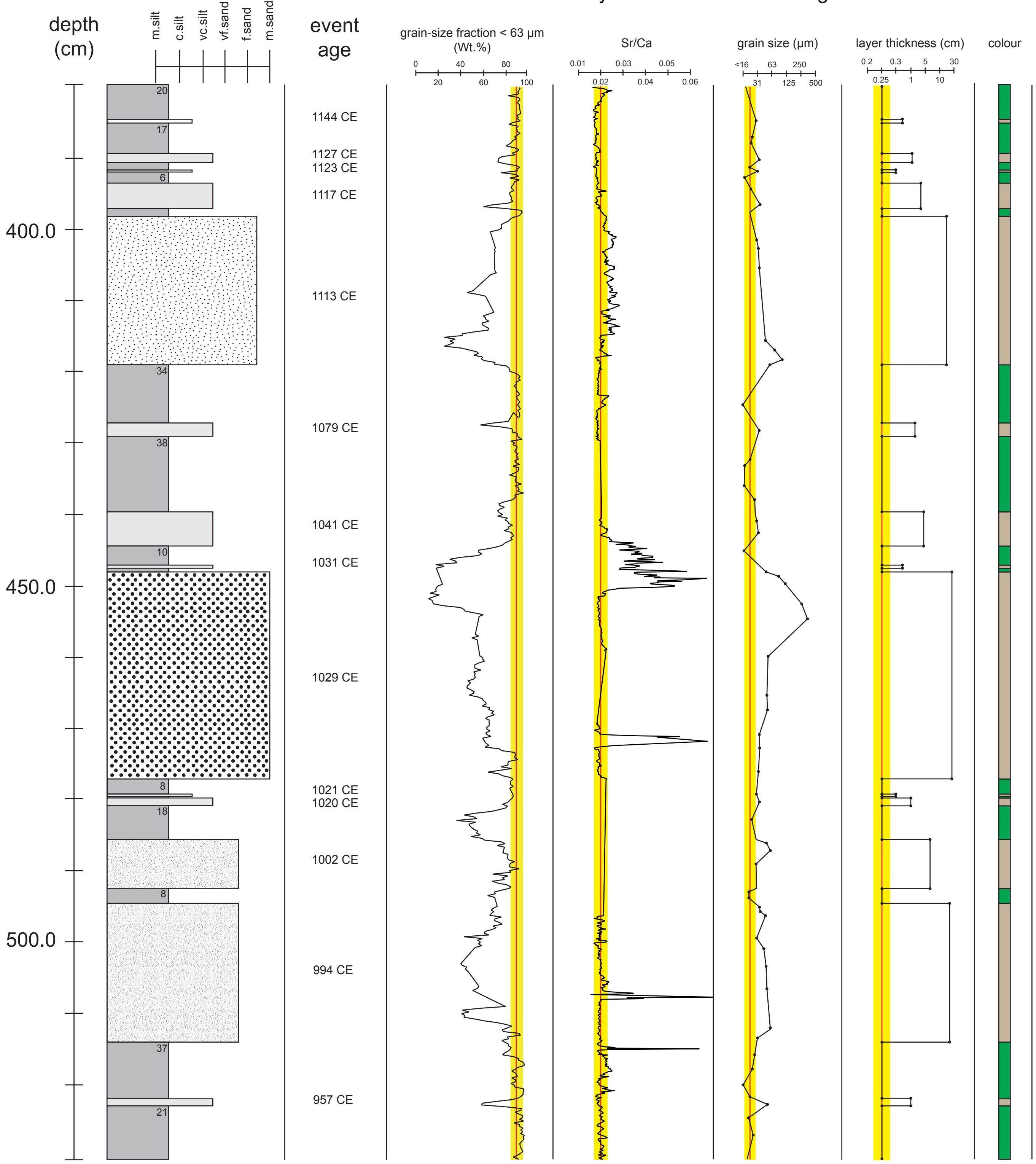

TC event layer identification: core log BH 6/3
depth (cm)
m.silt
c.silt
vc.silt
vf.sand
f.sand
m.sand
event age
grain-size fraction < 63 µm (Wt.%)
Sr/Ca
grain size (µm)
layer thickness (cm)
colour
400.0
450.0
500.0
1144 CE
1127 CE
1123 CE
1117 CE
1113 CE
1079 CE
1041 CE
1031 CE
1029 CE
1021 CE
1020 CE
1002 CE
994 CE
957 CE

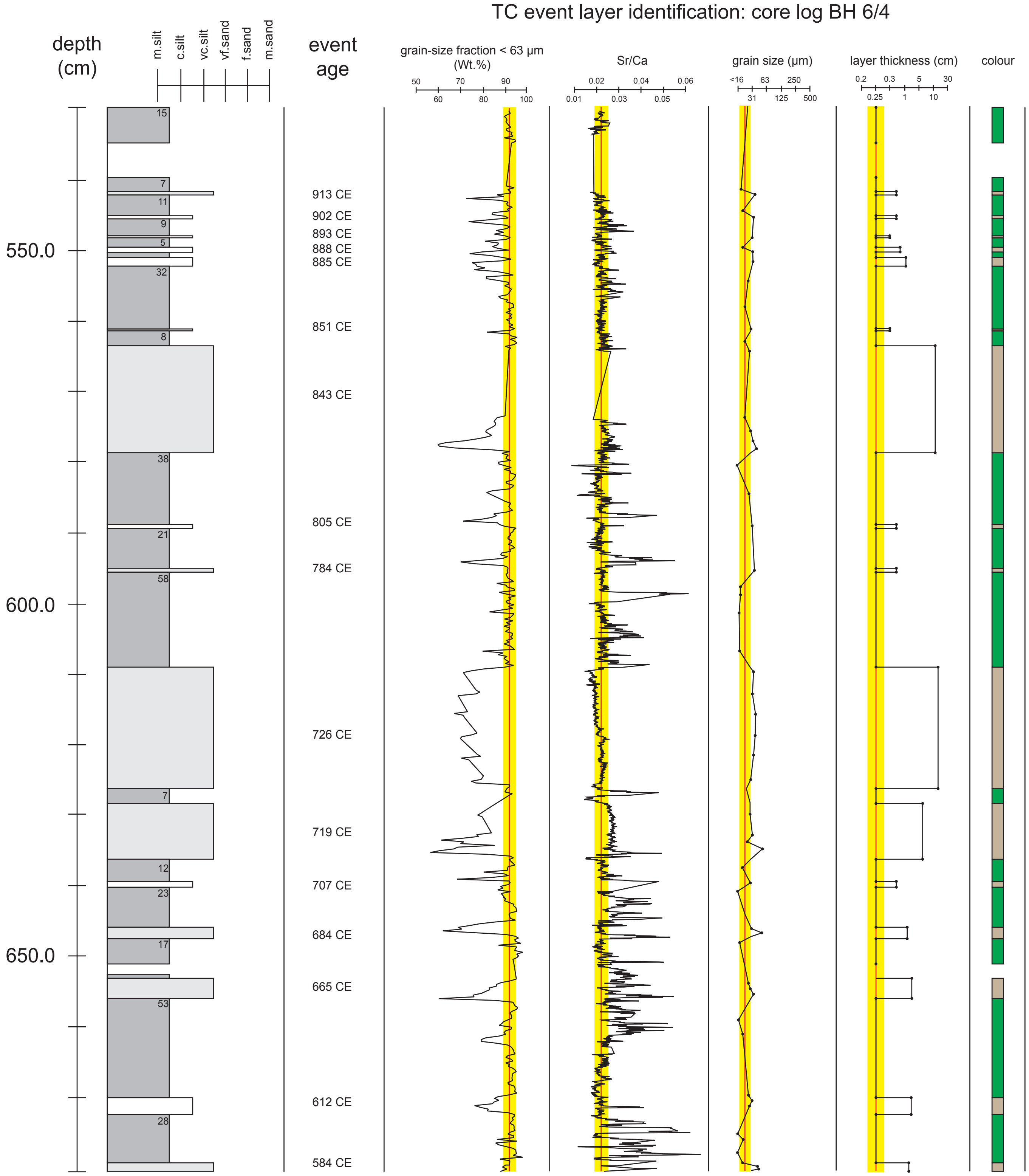
TC event layer identification: core log BH 6/4
depth (cm)
m.silt
c.silt
vc.silt
vf.sand
f.sand
m.sand
event age
grain-size fraction < 63 µm (Wt.%)
Sr/Ca
grain size (µm)
layer thickness (cm)
colour
550.0
600.0
650.0
913 CE
902 CE
893 CE
888 CE
885 CE
851 CE
843 CE
805 CE
784 CE
726 CE
719 CE
707 CE
684 CE
665 CE
612 CE
584 CE

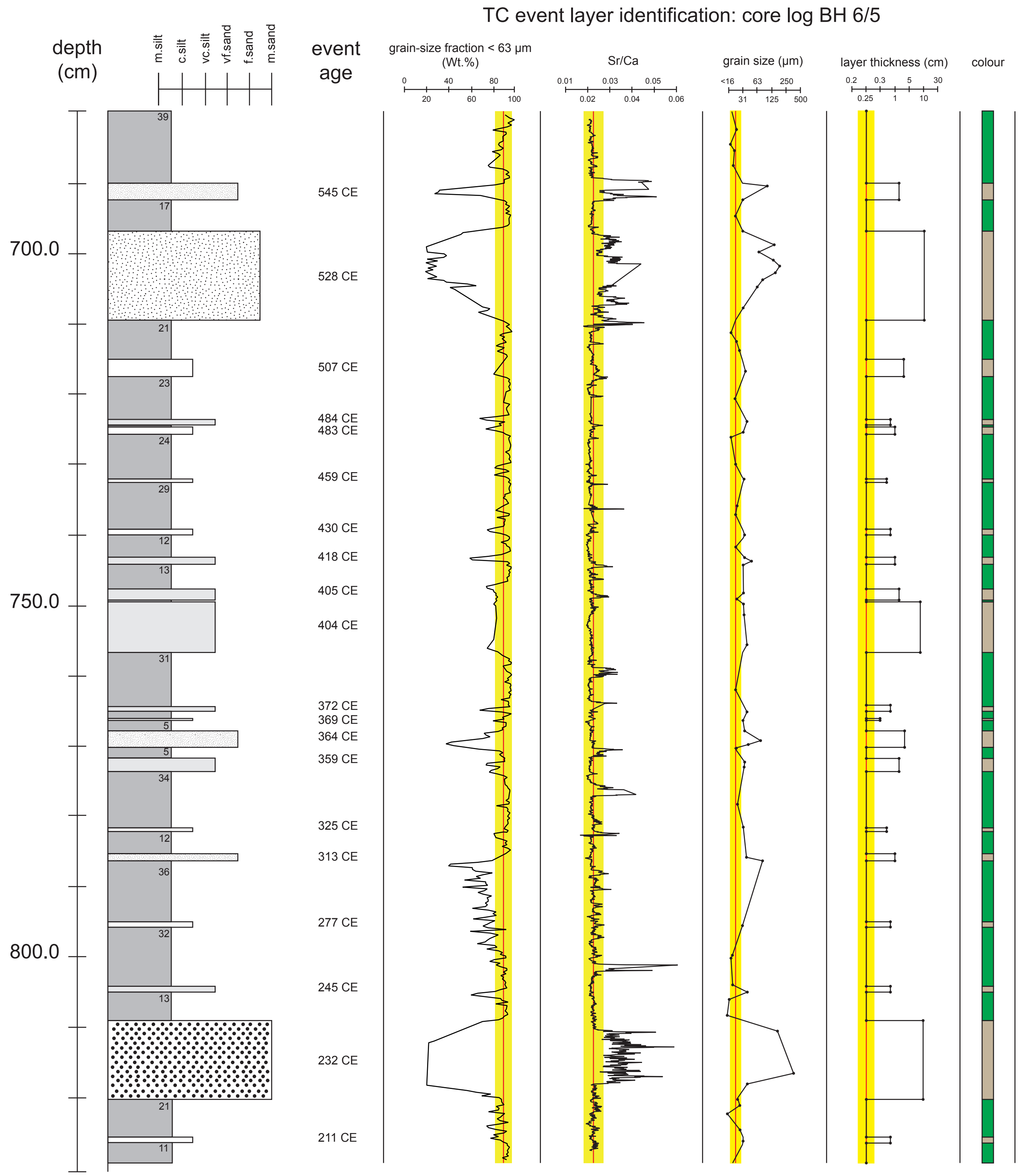
TC event layer identification: core log BH 6/5
depth (cm)
700.0
750.0
800.0
m.silt
c.silt
vc.silt
vf.sand
f.sand
m.sand
event age
545 CE
528 CE
507 CE
484 CE
483 CE
459 CE
430 CE
418 CE
405 CE
404 CE
372 CE
369 CE
364 CE
359 CE
325 CE
313 CE
277 CE
245 CE
232 CE
211 CE
grain-size fraction < 63 µm (Wt.%)
Sr/Ca
grain size (µm)
layer thickness (cm)
colour

TC event layer identification: core log BH 6/6

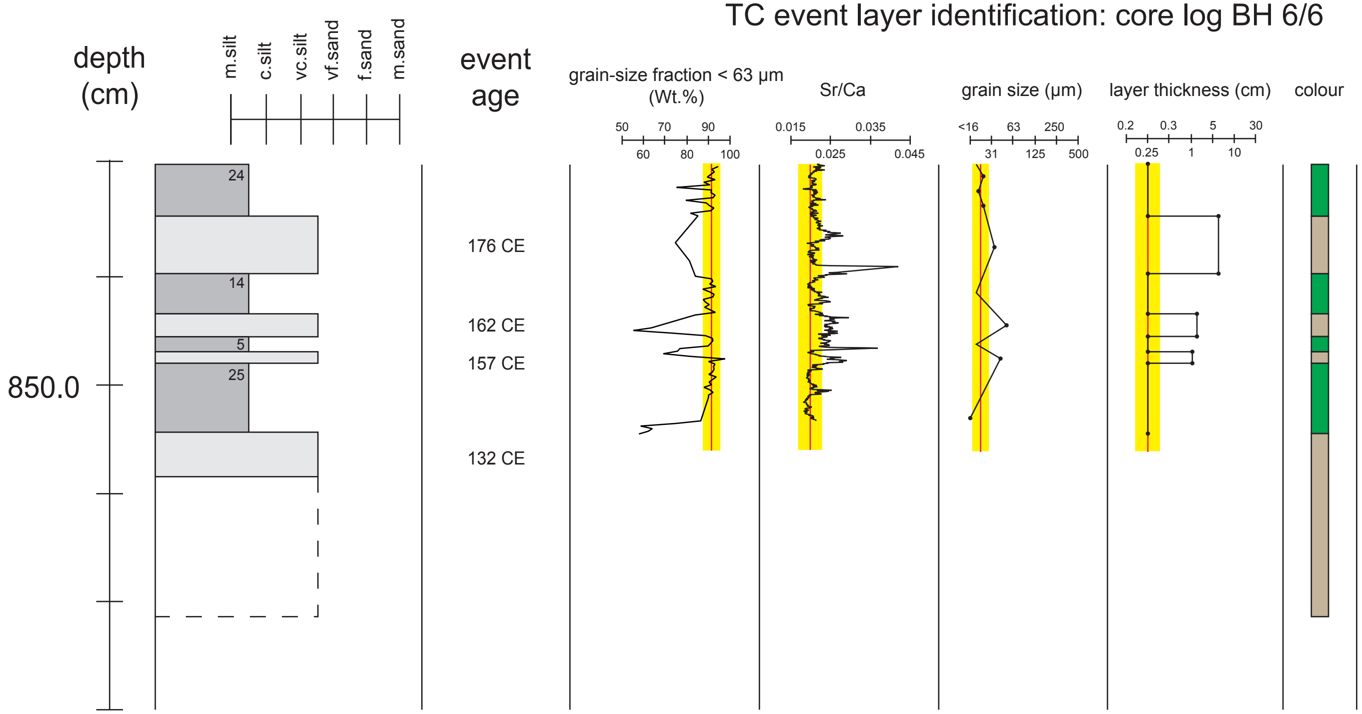

Suppl. 2: Correlation of historical TC record (1850 CE to 2017 CE), passing within 100 km radius around Lighthouse Reef, and proxy based event layer detection in core BH6.

Suppl. 3: a) The conceptual figure shows the genesis regions (shaded areas) of the four main Caribbean TC clusters and all TC reconstruction sites mentioned in the manuscript. **b,c,d,e)** Major landfall sites of the four clusters are highlighted with coloured star symbols. The red pin illustrates the position of Lighthouse Reef (presented TC record). The map was produced by digitising open access NASA satellite images with the vector graphic software Adobe Illustrator CS4 V.14.0. We acknowledge the use of imagery from the NASA Worldview application (https://worldview.earthdata.nasa.gov), part of the NASA Earth Observing System Data and Information System (EOSDIS). The simplified presentation of the TC clusters is based on the original data of Kossin et al. (2010).

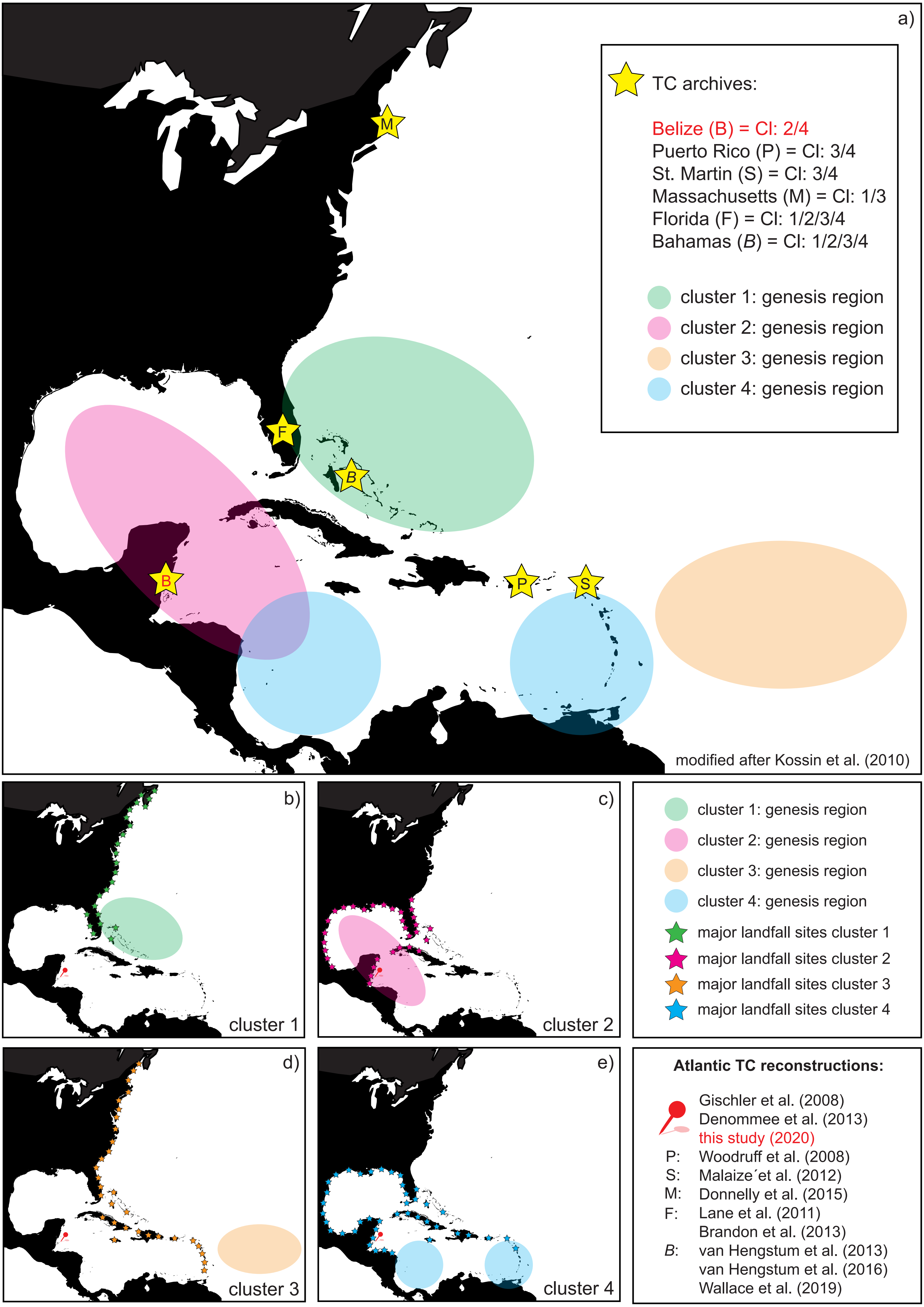
a)
TC archives:
Belize (B) = CI: 2/4
Puerto Rico (P) = CI: 3/4
St. Martin (S) = CI: 3/4
Massachusetts (M) = CI: 1/3
Florida (F) = CI: 1/2/3/4
Bahamas (*B*) = CI: 1/2/3/4
cluster 1: genesis region
cluster 2: genesis region
cluster 3: genesis region
cluster 4: genesis region
M
F
B
B
P
S
modified after Kossin et al. (2010)
b)
cluster 1
c)
cluster 2
cluster 1: genesis region
cluster 2: genesis region
cluster 3: genesis region
cluster 4: genesis region
major landfall sites cluster 1
major landfall sites cluster 2
major landfall sites cluster 3
major landfall sites cluster 4
d)
cluster 3
e)
cluster 4
Atlantic TC reconstructions:
Gischler et al. (2008)
Denommee et al. (2013)
this study (2020)
P: Woodruff et al. (2008)
S: Malaize´et al. (2012)
M: Donnelly et al. (2015)
F: Lane et al. (2011)
Brandon et al. (2013)
B: van Hengstum et al. (2013)
van Hengstum et al. (2016)
Wallace et al. (2019)

Suppl. 4: Seven detailed core logs in chronological order (BH7/1, BH6/1, BH6/2, BH6/3, BH6/4, BH6/5, BH6/6) with lithological and stratigraphic description (varve counting, TC event layer age, macro-fossil identification and sedimentary structures).

core log Blue Hole 7/1 (2017)

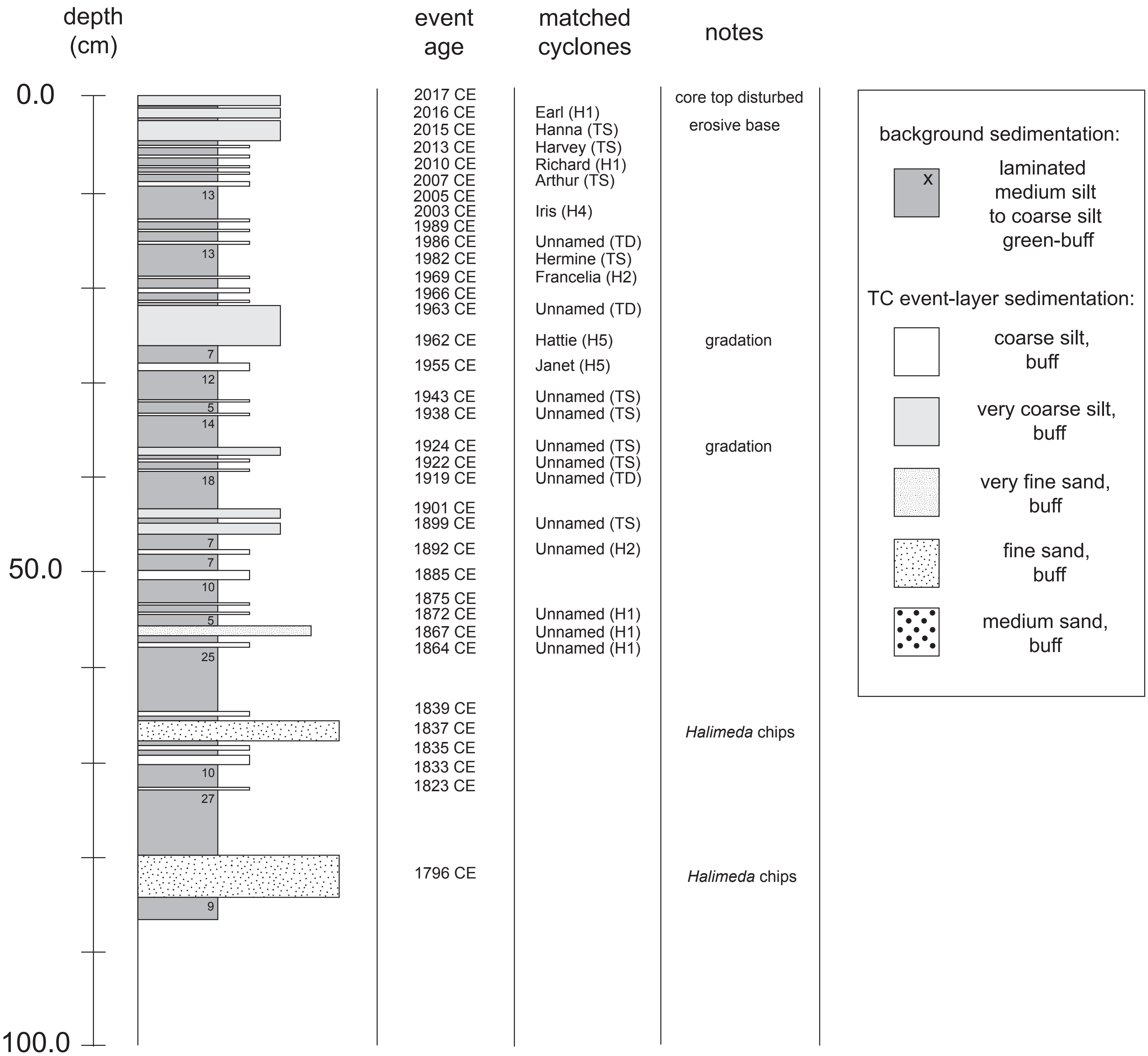

core log Blue Hole 6/1 (2017)

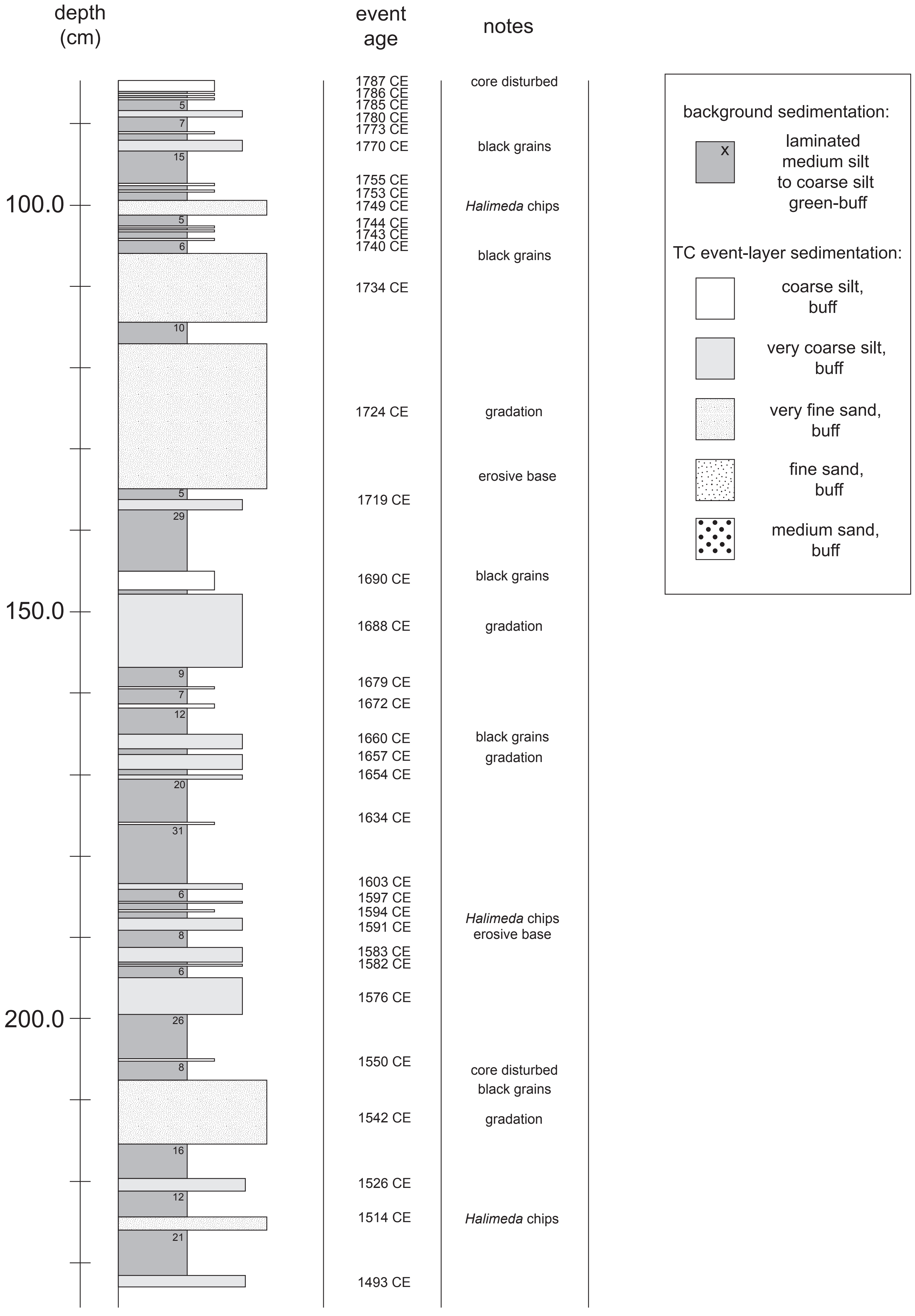

core log Blue Hole 6/2 (2017)

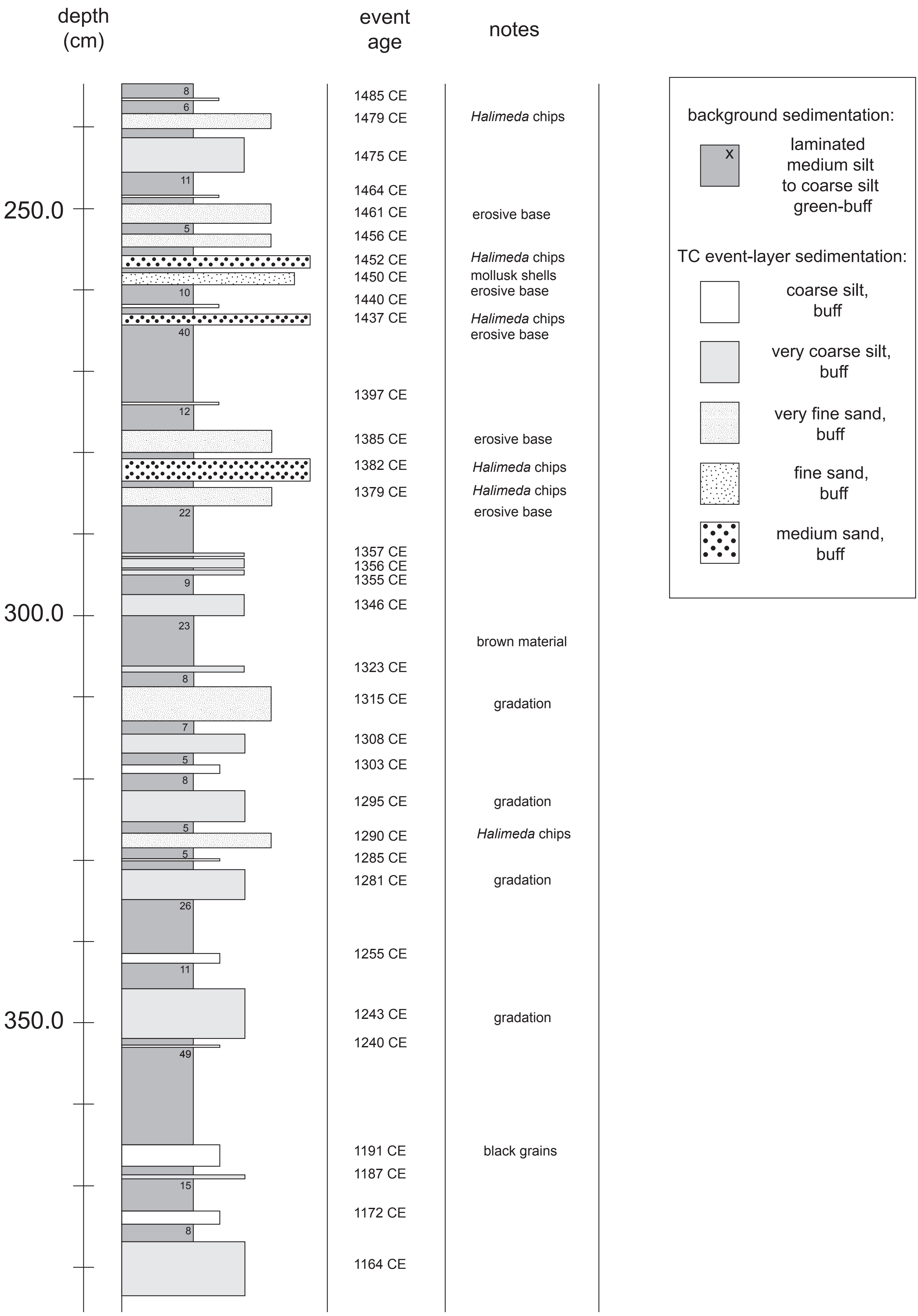

core log Blue Hole 6/3 (2017)

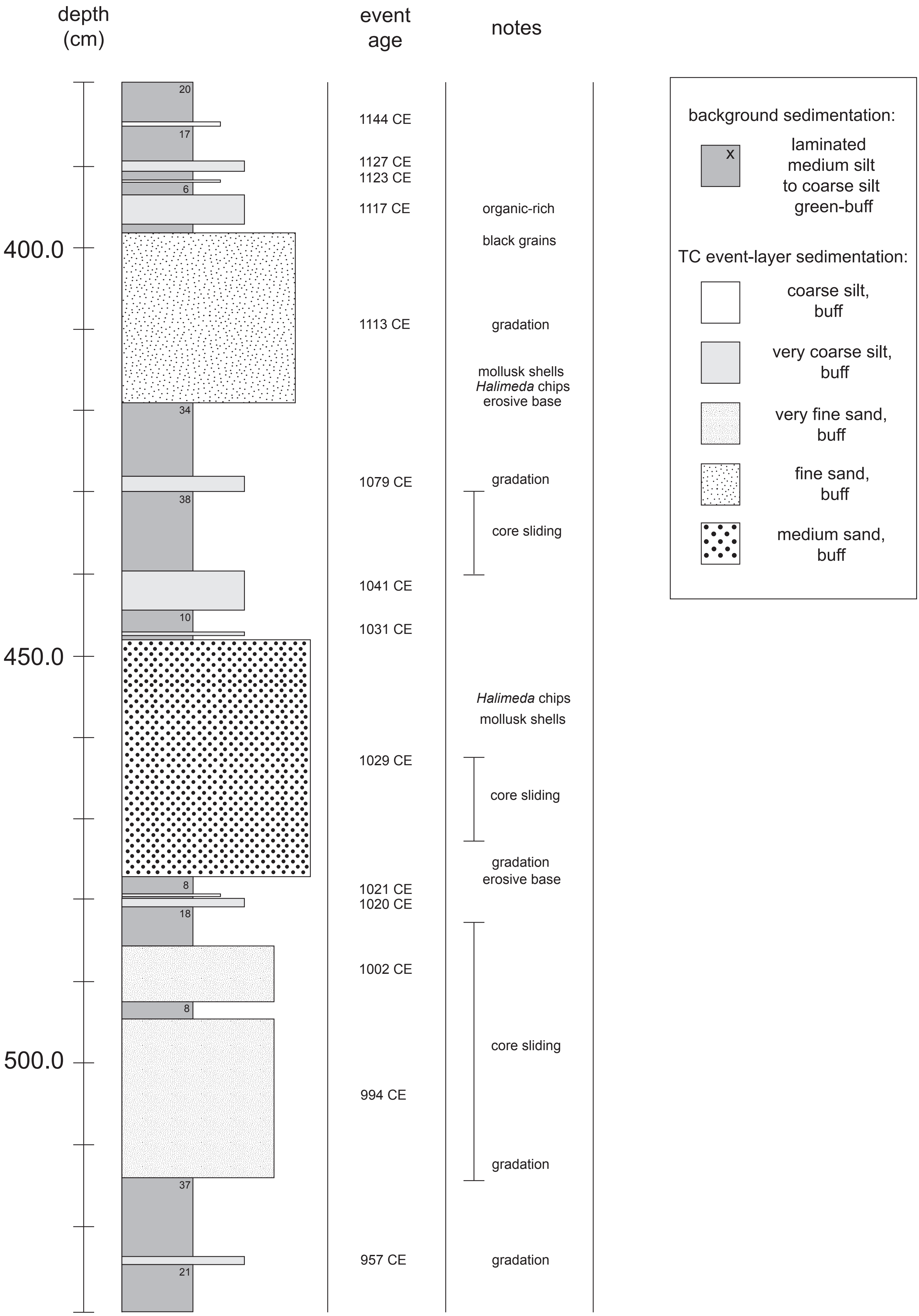

core log Blue Hole 6/4 (2017)

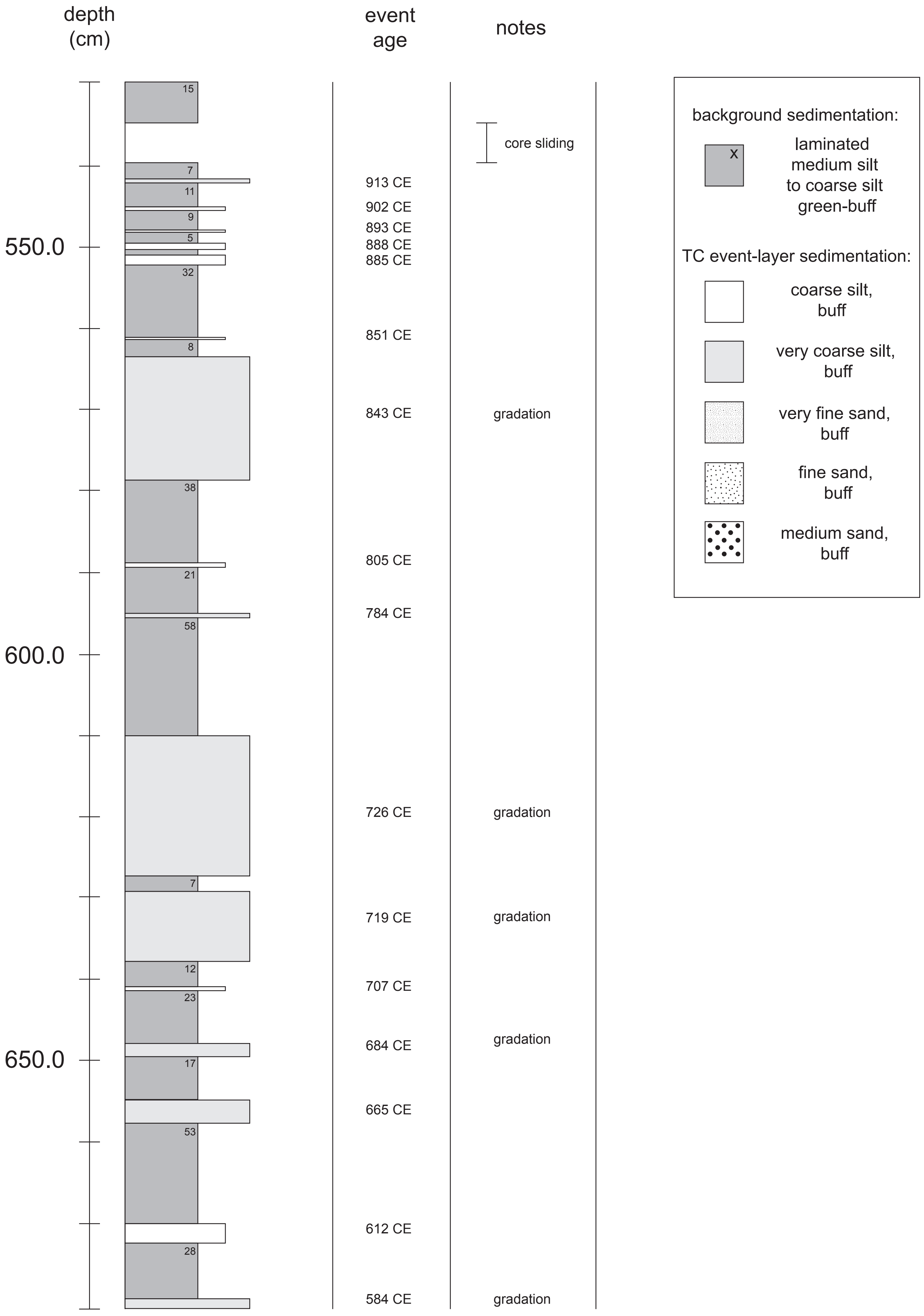

core log Blue Hole 6/5 (2017)

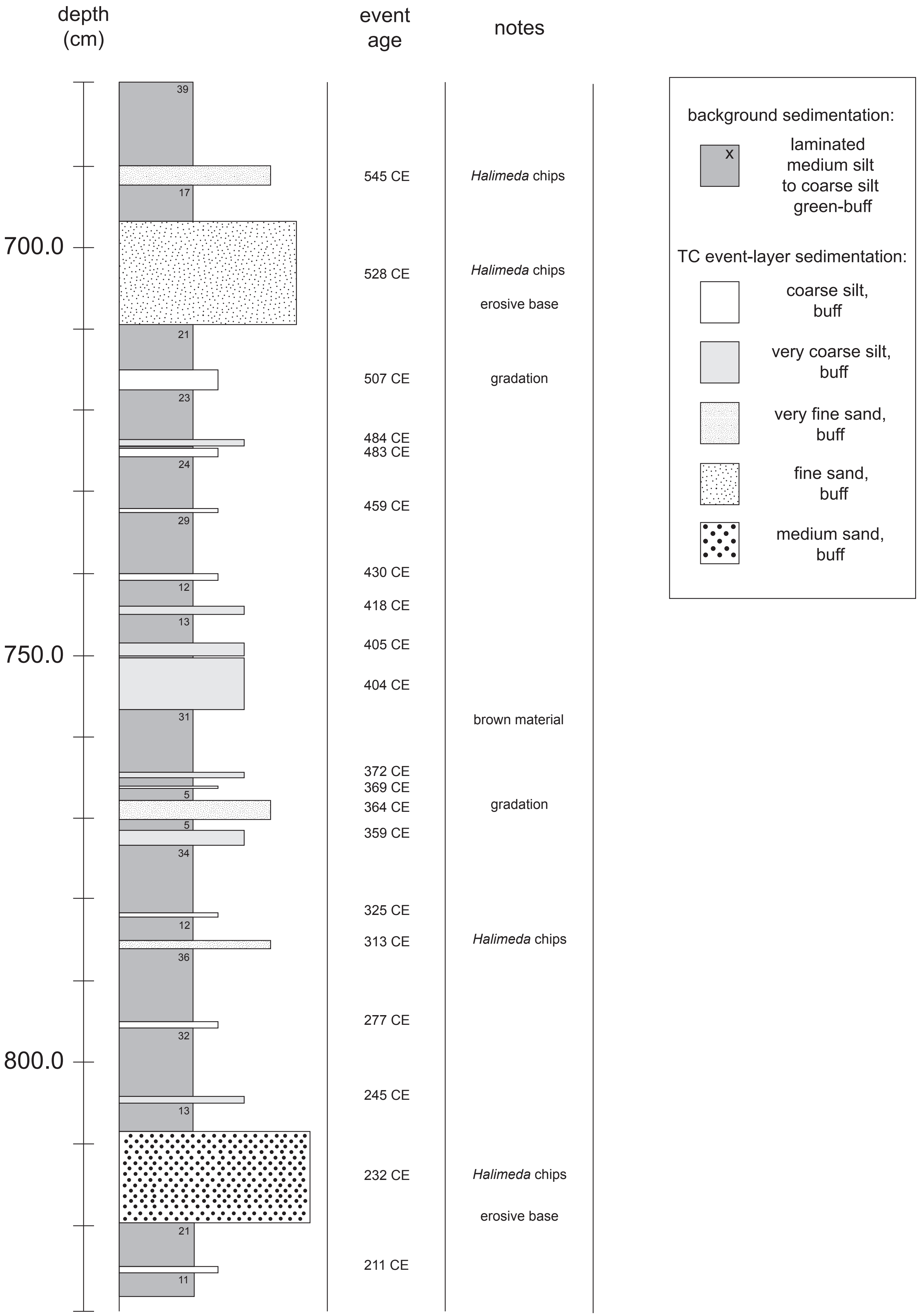

core log Blue Hole 6/6 (2017)

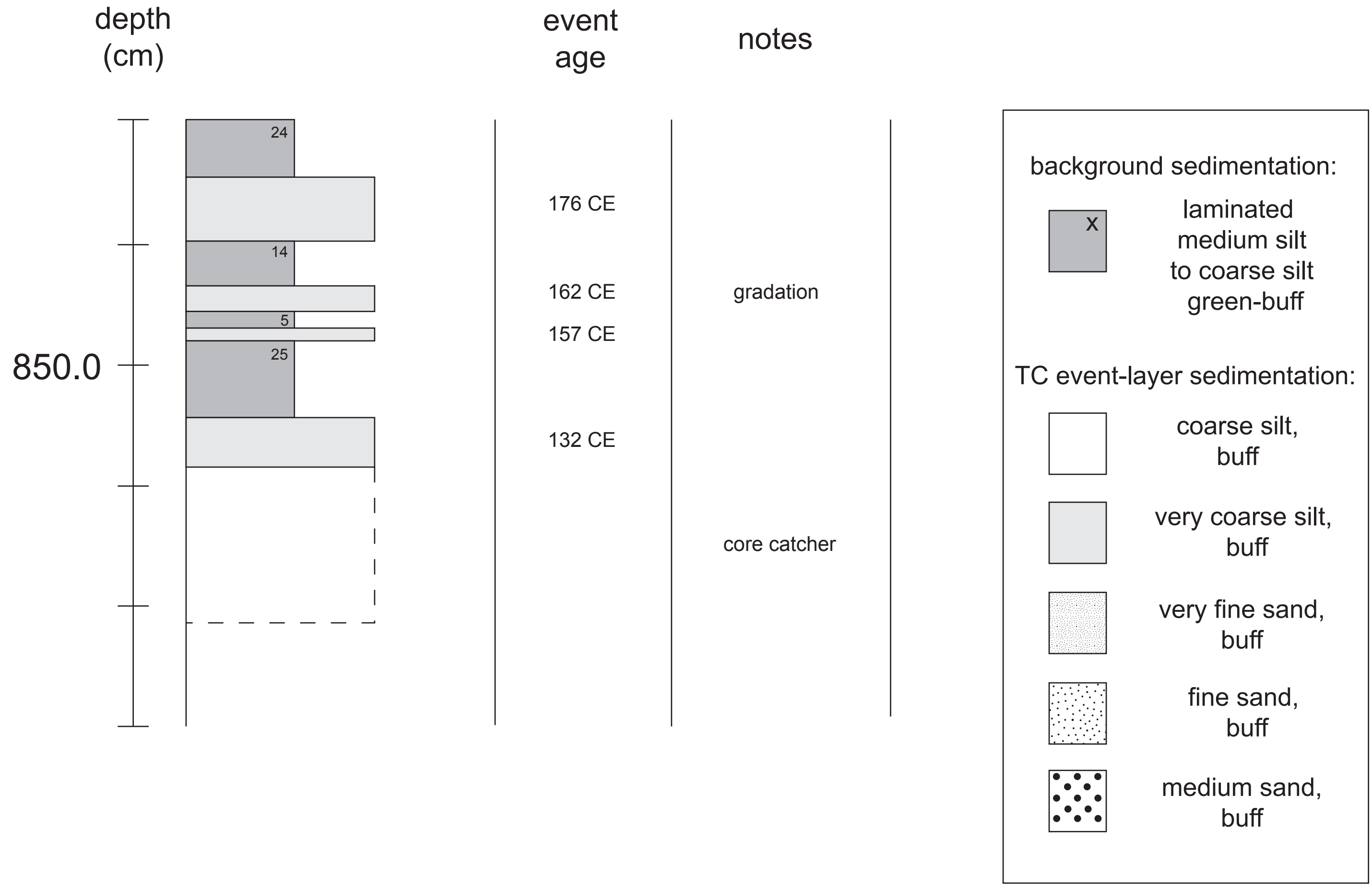

Suppl. 5: Raw data of textural analysis (sieving results < 2mm-63 μm).

Suppl. 6: Raw data for age (year) to core depth (mm) calibration.

Suppl. 7: ^{14}C AMS radiocarbon geochronology and comparison with varve counting results.

Suppl. 8: Raw data of grain size and sorting calculations including the grain sizes <63 μm (Laser Particle Size Analyser).

Suppl. 9: Raw data of XRF measurements (element content in raw counts).

Suppl. 10: Raw data of TC counts, event layer thickness and grain size.

Supplementary Information is linked to the online version of the paper at www.nature.com/srep/.
